# Posttraumatic Stress Disorder (PTSD) NAD/Sirtuin Deficiency and SARM1-Mediated Synaptic Vulnerability: Evidence for Accelerated Brain Aging Subtypes

**DOI:** 10.7759/cureus.110755

**Published:** 2026-06-12

**Authors:** Ngo Cheung

**Affiliations:** 1 Psychiatry, Cheung Ngo Medical Limited, Hong Kong, HKG

**Keywords:** ageing, nad, ptsd, sarm1, sirtuin, trauma

## Abstract

Anxiety disorders and posttraumatic stress disorder (PTSD) often occur alongside signs of accelerated biological aging, yet the molecular pathways that connect genetic liability to synaptic and circuit-level dysfunction remain unclear. We performed a multi-gene-set transcriptome-wide association study using large-scale genome-wide association study (GWAS) summary statistics for anxiety disorders and PTSD, with a focus on curated aging-related pathways across brain regions involved in fear, reward, memory, and stress regulation.

The analysis identified 185 significant enrichments, with strong cross-disorder concordance in senescence, telomere maintenance, and mitochondrial modules. PTSD showed a distinctive negative signature in nicotinamide adenine dinucleotide (NAD) metabolism and sirtuin-related pathways, most prominently driven by sirtuin 3 (SIRT3), together with evidence of sterile alpha and Toll/interleukin-1 receptor motif-containing 1 (SARM1)-linked axonal and synaptic vulnerability. Anxiety disorders showed stronger enrichment in mitochondrial intrinsic apoptosis and inflammatory-redox signaling, alongside glutamatergic and astrocytic plasticity changes. Both conditions shared senescence and deoxyribonucleic acid (DNA)-damage programs, with NIMA-related kinase 4 (NEK4) emerging as a common driver. These pathways converged on complement-mediated pruning and presynaptic remodeling, suggesting a mechanistic bridge from cellular aging to fear and reward circuit dysfunction.

Together, the findings support an association-based model in which genetic liability for anxiety disorders and PTSD converges on stress-aging biology at the synapse, while diverging by disorder. This framework points to candidate biologically distinct subtypes and offers practical opportunities for biomarker development, patient stratification, and subtype-guided therapeutic strategies. These subtype labels are proposed biological hypotheses rather than validated clinical categories.

## Introduction

Anxiety disorders and posttraumatic stress disorder (PTSD) are common and disabling conditions that affect large numbers of people worldwide. Their burden is not limited to fear, worry, avoidance, intrusive memories, or hyperarousal. Both conditions are also associated with medical comorbidities that are typically linked to aging, including cardiovascular disease, metabolic dysfunction, cognitive decline, and chronic inflammation [1-3]. A growing body of work also suggests that people with anxiety disorders or PTSD show markers of accelerated biological aging, including shorter leukocyte telomere length, epigenetic age acceleration, and higher allostatic load [4-7]. These findings suggest that the biology of these disorders may extend beyond acute fear and stress responses and involve deeper disturbances in cellular aging pathways.

Psychiatric genetics has made important progress in identifying risk loci and broad biological signals for anxiety disorders and PTSD through genome-wide association studies (GWAS) [8-10]. Still, much of the field remains focused on individual loci or general inflammatory pathways. These approaches are useful, but they often leave a gap between genetic association and mechanism. In particular, they do not fully explain how genetic risk might affect synaptic function, circuit plasticity, and cellular aging programs in brain tissue. This gap matters because anxiety disorders and PTSD share clinical and neurobiological features, yet they also differ in trauma-related onset, symptom structure, and treatment response [11,12].

Transcriptome-wide association studies (TWAS) provide one way to bridge this gap. TWAS methods use expression quantitative trait loci (eQTLs) to estimate genetically predicted gene expression in disease-relevant tissues and then test whether that predicted expression is associated with disease risk [13-16]. When applied to brain regions involved in fear, reward, memory, and executive control--such as the caudate, nucleus accumbens, hippocampus, amygdala, frontal cortex, and anterior cingulate cortex--this approach can highlight functional pathways that may be altered by genetic liability. TWAS is especially useful for studying aging biology because major hallmarks of aging, including mitochondrial dysfunction, cellular senescence, altered nicotinamide adenine dinucleotide (NAD) metabolism, and inflammatory signaling, leave measurable signatures in gene-expression programs [17,18].

Here, “accelerated brain aging subtypes” refers to biologically defined profiles in which genetically predicted expression patterns resemble aging-related programs in brain tissue, such as NAD/sirtuin deficiency, senescence/telomere stress, mitochondrial-apoptotic vulnerability, or complement-pruning activation. The term is used as a mechanistic stratification hypothesis, not as a clinical diagnosis.

We hypothesized that genetic liability for anxiety disorders and PTSD would converge on stress-aging biology. Specifically, we expected enrichment of pathways involving mitochondrial and NAD-related stress, deoxyribonucleic acid (DNA) damage, senescence, apoptosis, inflammatory signaling, and downstream effects on synaptic pruning and plasticity. At the same time, we expected the two disorders to diverge. PTSD risk was expected to show stronger signatures of NAD/sirtuin suppression and axonal or synaptic vulnerability, whereas anxiety risk was expected to emphasize mitochondrial apoptosis thresholds and inflammatory-glutamatergic plasticity. This model could help explain why both conditions show accelerated aging phenotypes while still producing different clinical patterns. It also links molecular findings to known circuit-level mechanisms involving fear generalization, extinction learning, reward processing, and threat regulation [19-22].

To test this model, we performed a multi-gene-set TWAS and enrichment analysis using summary statistics from two large European-ancestry GWAS: one for major anxiety disorders and one for PTSD. The analysis focused on curated aging-related gene sets. The goals were to identify enriched aging pathways and their main gene drivers in brain tissue, develop a mechanistic model linking these pathways to synaptic and circuit dysfunction, and derive translational implications for biomarkers, patient stratification, and therapeutic hypotheses. The primary objective was descriptive and comparative: to test whether curated aging-related pathways were enriched in anxiety disorders and PTSD and whether the two disorders showed shared or divergent profiles. Downstream mechanistic and translational interpretations were treated as hypotheses generated by these enrichment patterns.

This work builds on several related areas. The hallmarks of the aging framework describe interconnected processes such as mitochondrial dysfunction, cellular senescence, and chronic inflammation that contribute to organismal decline [17,18]. Programmed axon degeneration, mediated in part by the NAD-consuming enzyme sterile alpha and Toll/interleukin-1 receptor motif-containing 1 (SARM1), provides a plausible link between metabolic stress and synaptic disconnection [23-25]. Complement-mediated synaptic pruning, first described as a developmental process, can become maladaptive in adult brains under inflammatory or stress-related conditions [26-28]. Finally, the neurocircuitry of fear conditioning and extinction, centered on the amygdala, hippocampus, and medial prefrontal cortex, provides a framework for understanding how molecular changes may translate into persistent threat memory, impaired safety learning, and avoidance behavior [20-22].

## Materials and methods

Genome-wide association study data sources

We used publicly available summary statistics from two large-scale GWAS. The anxiety disorders analysis was based on a study by Strom et al. [10], which included 122,341 European-ancestry cases with major anxiety disorders and matched controls. The PTSD analysis used data from a study by Nievergelt et al. [9], comprising European-ancestry participants with PTSD diagnoses. Both datasets had undergone standard quality control, imputation, and association testing procedures as described in the original publications.

TWAS and gene-set enrichment pipeline

Genetically predicted gene expression was imputed using S-PrediXcan from the MetaXcan framework [13,14], with brain tissue-specific eQTL weights from Genotype-Tissue Expression Project version 8 (GTEx v8) [15]. We focused on the caudate basal ganglia, nucleus accumbens basal ganglia, hippocampus, amygdala, frontal cortex Brodmann area 9 (BA9), and anterior cingulate cortex Brodmann area 24 (BA24). These regions were selected because of their established roles in fear processing, reward, memory, and executive regulation.

Multi-gene-set enrichment analyses were conducted using curated aging-related gene sets from Reactome, Kyoto Encyclopedia of Genes and Genomes (KEGG), Hallmark, Gene Ontology, and WikiPathways as represented in Molecular Signatures Database-derived collections [16]. The collections included cellular senescence, telomere maintenance, DNA damage response, intrinsic and extrinsic apoptosis, NAD biosynthesis and metabolism, sirtuin-related processes, mitochondrial function and oxidative phosphorylation, mitophagy, complement and innate immune activation, microglial activation, synaptic plasticity regulation, neurotransmitter release, glutamatergic and dopaminergic signaling, and insulin/glucagon-like peptide-1 (GLP-1)-related metabolic pathways.

Gene identifiers were harmonized before gene-set assignment, duplicate genes within a set were removed, and only genes with available TWAS statistics in the relevant tissue were retained. The archived analysis output used the multi-gene-set mode and was generated on May 13, 2026.

Statistical thresholds and filtering

Enrichment was evaluated using Stouffer’s Z-score combination across genes within each set. We applied a nominal significance threshold of raw p < 0.05 and absolute Stouffer Z > 1.96. Additional filters included false discovery rate (FDR) correction where available, permutation-based p-values, Wilcoxon rank-sum tests, absolute Cohen’s d > 0.5, sign-concordance rate > 0.7, Kendall’s tau > 0.3, Pearson r > 0.3, Lin’s concordance correlation coefficient (CCC) > 0.5, and rank-biserial r > 0.3. Leave-one-out influence analysis was used to identify genes that disproportionately drove set-level signals. Results were retained only when they met at least two independent statistical criteria, such as a significant permutation p-value and the Stouffer Z threshold, to reduce false positives.

This multi-criterion thresholding scheme was used because the analysis combined small, medium, and large gene sets, and no single test was expected to capture all robust pathway patterns. Stouffer Z and nominal p-values screened for pathway-level signal; permutation and Wilcoxon tests assessed robustness to gene-level distributional assumptions; effect-size and concordance filters emphasized consistent direction or meaningful magnitude; and FDR summarized multiple-testing control where available. Overlapping or correlated pathways were not pruned before testing because several aging pathways are biologically nested. Therefore, related pathway hits were interpreted as families of convergent signals rather than as statistically independent discoveries. No pathway family survived the most stringent family-wise FDR summary, so the results are presented as prioritized enrichment signals requiring replication rather than definitive family-wise discoveries.

## Results

Overview of significant enrichments

The multi-gene-set enrichment analysis identified 185 findings that met the pre-specified statistical criteria across the curated aging-related gene sets. Because no pathway family survived the most stringent family-wise FDR screen across the full analysis, the following results should be read as convergent, prioritized enrichment signals rather than final proof of pathway activation. Several core biological modules showed high concordance between anxiety disorders and PTSD (Table 1). Pearson correlations of gene-level Z-scores between the two disorder profiles reached r = 0.60 for cellular senescence gene sets and r = 0.70 for telomere maintenance sets. Lin’s concordance correlation coefficients exceeded 0.69 in both modules. Mitochondrial and oxidative phosphorylation modules showed more moderate but still significant similarity, with correlations of about 0.45 to 0.50.

**Table 1:** Cross-Disorder Concordance in Core Aging-Related Modules (ptsd vs. anx_cc) Note: r = Pearson correlation coefficient; ρ = Spearman rank correlation coefficient; CCC = Lin's concordance correlation coefficient; τ = Kendall's tau; Sign = sign-concordance rate. All reported Pearson and Spearman values were significant at p < .05. PTSD = posttraumatic stress disorder; anx_cc = anxiety disorder case-control phenotype; HLA = human leukocyte antigen; GOBP = Gene Ontology Biological Process; REACTOME = Reactome pathway database; HALLMARK = Molecular Signatures Database Hallmark gene set; MOOTHA = Mootha mitochondrial gene set; n = number of genes.

Module / Gene Set	n	r	ρ	CCC	τ	Sign
HLA complex	16	.89	.87	.87	.67	.88
GOBP regulation of cellular senescence	35	.76	.63	.73	.47	.74
REACTOME telomere maintenance	54	.70	.60	.70	.43	.64
GOBP cellular senescence	75	.60	.54	.57	.39	.65
GOBP regulation of synaptic plasticity	151	.60	.51	.59	.37	.71
HALLMARK apoptosis	131	.51	.52	.51	.37	.68
MOOTHA mitochondria	353	.50	.40	.50	.28	.65
HALLMARK oxidative phosphorylation	149	.45	.40	-	-	-
GOBP intrinsic apoptotic signaling	264	.47	.44	.47	.31	.64

Many of the strongest signals are localized to brain regions with established roles in emotion regulation and reward processing. Enrichment in the caudate and nucleus accumbens basal ganglia was especially consistent for apoptosis, senescence, and synaptic plasticity sets. Hippocampal and amygdala signals were prominent for DNA-damage response and inflammatory pathways. These regional patterns are consistent with the involvement of striatal and limbic circuits in both anxiety disorders and PTSD.

PTSD-specific negative NAD/sirtuin/SIRT3 signature

A clear divergence between PTSD and anxiety disorders emerged in NAD metabolism and sirtuin-related gene sets. PTSD showed consistent negative enrichment across several NAD biosynthetic and sirtuin-associated collections (Table 2). The WP NAD biosynthetic pathways "ALL" set yielded a Stouffer Z of -5.88, with permutation p = 0.015. Tissue-specific values were -2.96 in the anterior cingulate cortex, -2.95 in the amygdala, and -3.42 in the hippocampus. By contrast, anxiety disorders showed positive enrichment in overlapping nicotinate and nicotinamide metabolism sets, with an overall Z of +4.19.

**Table 2:** PTSD-Specific NAD/Sirtuin Signature and Leading SIRT3 Driver Note. Z = Stouffer's combined Z-score; p = permutation or Wilcoxon p-value, as reported; % = leave-one-out influence on pooled pathway Z. PTSD = posttraumatic stress disorder; anx_cc = anxiety disorder case-control phenotype; WP = WikiPathways; NAD = nicotinamide adenine dinucleotide; NAD⁺ = oxidized nicotinamide adenine dinucleotide; SIRT1 = sirtuin 1; SIRT3 = sirtuin 3; ACC = anterior cingulate cortex; BA24 = Brodmann area 24; KEGG = Kyoto Encyclopedia of Genes and Genomes; GOMF = Gene Ontology Molecular Function; NAPRT = nicotinate phosphoribosyltransferase; NMNAT2 = nicotinamide nucleotide adenylyltransferase 2; n = number of genes.

Gene Set	Trait	Tissue	n	Z	p
WP NAD biosynthetic pathways	ptsd	Pooled	19	-5.88	.015
WP NAD biosynthetic pathways	ptsd	Hippocampus	13	-3.42	.048
WP NAD biosynthetic pathways	ptsd	ACC (BA24)	17	-2.96	.020
WP NAD biosynthetic pathways	ptsd	Amygdala	14	-2.95	.025
WANG adipogenic genes repressed by SIRT1	ptsd	Pooled	18	-4.98	.041
KEGG nicotinate and nicotinamide metabolism	anx_cc	Pooled	22	+4.19	.030
Leading leave-one-out drivers (PTSD)					
GOMF NAD⁺ binding--SIRT3	ptsd	-	-	-7.58	72.9%
WP NAD metabolism/sirtuins/aging--SIRT3	ptsd	-	-	-7.58	109.0%
REACTOME nicotinate metabolism--NAPRT	ptsd	-	-	-3.74	-
KEGG nicotinate/nicotinamide--NMNAT2	ptsd	-	-	+2.62	-

Sirtuin 3 (SIRT3), a mitochondrial deacetylase, was the most influential driver in these PTSD-associated sets. Across GOMF NAD⁺ binding, WP NAD biosynthetic pathways, and WP NAD metabolism/sirtuins/aging collections (SIRT3) showed Z = -7.58 and accounted for up to 72.9% of the pathway signal in leave-one-out analyses. Other notable drivers included nicotinamide nucleotide adenylyltransferase 2 (NMNAT2), with Z = +2.62 in PTSD; nicotinate phosphoribosyltransferase (NAPRT), with Z = -3.74 in PTSD compared with +2.30 in anxiety; and several poly(adenosine diphosphate-ribose) polymerase (PARP) family members in related NAD-binding collections. Differential testing confirmed that the WANG adipogenic genes repressed by SIRT1 ALL set were significantly more negative in PTSD than in anxiety, with a mean Z difference of -1.87, Cohen’s d = -0.74, and FDR-corrected p = 0.035. Overall, these findings are consistent with lower genetically predicted NAD/sirtuin-related pathway tone in PTSD, particularly in mitochondrial compartments, but they do not directly measure tissue NAD levels or sirtuin enzymatic activity.

NAD-SARM1-synaptic/axon-vulnerability axis

Integration across NAD metabolism, mitophagy, and pruning-related gene sets suggested a possible link between metabolic stress and axonal or synaptic vulnerability. SARM1, a key regulator of programmed axon degeneration, showed positive enrichment in both disorders within relevant mitophagy and pruning collections, with Z values around +3.10 to +3.15 (Table 3). This signal occurred alongside negative NAD/sirtuin findings in PTSD and positive mitochondrial and apoptosis signals in anxiety.

**Table 3:** Aging-to-Pruning/Plasticity Bridge: Complement, Microglia, Plasticity, and Release Machinery Note. Direction reflects the sign of Stouffer's Z-score: ↑ = positive enrichment; ↓ = negative enrichment. PTSD = posttraumatic stress disorder; anx_cc = anxiety disorder case-control phenotype; REACTOME = Reactome pathway database; GOBP = Gene Ontology Biological Process; ADULT_MICRO = adult microglial gene signature; C4A = complement C4A; C1QA = complement C1q A chain; C1QC = complement C1q C chain; TREM1 = triggering receptor expressed on myeloid cells 1; TREM2 = triggering receptor expressed on myeloid cells 2; SARM1 = sterile alpha and Toll/interleukin-1 receptor motif-containing 1; MAP1A = microtubule-associated protein 1A; STAU1 = staufen double-stranded ribonucleic acid binding protein 1; ARC = activity-regulated cytoskeleton-associated protein; DLG4 = discs large membrane-associated guanylate kinase scaffold protein 4; STX1A = syntaxin 1A; VAMP2 = vesicle-associated membrane protein 2; GRM3 = glutamate metabotropic receptor 3; GRIA1 = glutamate ionotropic receptor α-amino-3-hydroxy-5-methyl-4-isoxazolepropionic acid type subunit 1.

Gene	Module	Trait	Z	Direction
C4A	REACTOME complement	anx_cc	+11.01	↑
C4A	REACTOME complement	ptsd	+12.33	↑
C1QA	GOBP synapse pruning	ptsd	-2.84	↓
C1QC	GOBP synapse pruning	anx_cc	+2.47	↑
TREM1	ADULT_MICRO	anx_cc	+5.54	↑
TREM2	GOBP synapse pruning	ptsd	+3.72	↑
SARM1	GOBP synapse pruning	anx_cc / ptsd	+3.10 / +3.15	↑
MAP1A	Regulation of synaptic plasticity	anx_cc / ptsd	-7.33 / -10.51	↓
STAU1	Regulation of synaptic plasticity	anx_cc / ptsd	-7.09 / -5.91	↓
ARC	Regulation of synaptic plasticity	anx_cc	-4.64	↓
DLG4	Regulation of synaptic plasticity	ptsd	-3.96	↓
STX1A	REACTOME neurotransmitter release	ptsd	-7.01	↓
VAMP2	REACTOME neurotransmitter release	anx_cc / ptsd	+6.98 / +3.57	↑
GRM3	GOBP glutamatergic	anx_cc	+7.36	↑
GRIA1	GOBP glutamatergic	ptsd	+5.35	↑

Leave-one-out analyses indicated that SARM1 contributed substantially to mitophagy enrichment in both conditions. The overall pattern is compatible with a model in which NAD depletion or sirtuin suppression lowers the threshold for SARM1 activation, increasing susceptibility to axon or synapse disconnection. This remains an inferred pathway-level link and does not demonstrate SARM1 activation in patient brain tissue. This interpretation is consistent with studies showing that SARM1 activation promotes axon degeneration through NAD+ destruction and with broader work on programmed axon degeneration as a pathway linking injury and disease [23-25,29,30]. The axis was further supported by concurrent enrichment in mitochondrial dynamics genes, including mitofusin 2 (MFN2), optic atrophy 1 (OPA1), and ras homolog family member T1 (RHOT1), and synaptic vesicle release machinery, suggesting a coordinated effect on presynaptic integrity.

Shared senescence/telomere/DNA-damage architecture

Both disorders showed strong and highly concordant enrichment in senescence, telomere maintenance, and DNA-damage response gene sets (Table 4). Pearson correlations of Z-scores within these modules ranged from 0.60 to 0.70, with Lin’s concordance correlation coefficients between 0.69 and 0.73. The GOBP CELLULAR SENESCENCE ALL set yielded r = 0.599, p < 0.001, and rho = 0.537, p = 1 x 10^-6, between anxiety and PTSD profiles.

**Table 4:** Shared Senescence, Telomere, and DNA-Damage Drivers Note. Δ = ΔStouffer, defined as the change in pathway-level Z-score with the gene removed; % = leave-one-out influence. PTSD = posttraumatic stress disorder; anx_cc = anxiety disorder case-control phenotype; GOBP = Gene Ontology Biological Process; REACTOME = Reactome pathway database; DNA = deoxyribonucleic acid; NEK4 = NIMA-related kinase 4; PPP6C = protein phosphatase 6 catalytic subunit; NBN = nibrin; ATM = ataxia telangiectasia mutated; POT1 = protection of telomeres 1; TERF1 = telomeric repeat binding factor 1; TERF2 = telomeric repeat binding factor 2; WRN = Werner syndrome RecQ-like helicase; RTEL1 = regulator of telomere elongation helicase 1.

Gene	Trait	Gene set	Z	Δ	%
NEK4	anx_cc	GOBP cellular senescence	+12.93	+1.48	100.5
NEK4	ptsd	GOBP cellular senescence	+7.8	+0.87	21.6
NEK4	anx_cc	Regulation of cellular senescence	+12.93	+2.15	97.8
NEK4	ptsd	Regulation of cellular senescence	+7.8	+1.3	56
PPP6C	anx_cc	REACTOME telomere	+9.25	+1.23	41.5
PPP6C	ptsd	REACTOME telomere	+11.46	+1.56	90.4
NBN	ptsd	REACTOME cellular senescence	+7.43	+0.74	115.1
ATM	ptsd	REACTOME cellular senescence	+6.3	+0.63	97.4
POT1	ptsd	REACTOME telomere	+7.00	+0.94	54.9
TERF1	ptsd	REACTOME telomere	+4.72	+0.63	36.8
TERF2	ptsd	REACTOME cellular senescence	-3.61	-0.36	56.6
WRN	ptsd	REACTOME telomere	+3.5	+0.47	27
RTEL1	ptsd	REACTOME telomere	+3.44	+0.46	26.5

Prominent driver genes included ataxia telangiectasia mutated (ATM), with Z = +6.30 in PTSD senescence and apoptosis sets; nibrin (NBN), with Z = +7.43; protection of telomeres 1 (POT1), with Z = +7.00 in PTSD telomere and senescence sets; telomeric repeat binding factor 1 (TERF1), with Z = +4.72; telomeric repeat binding factor 2 (TERF2), with Z = -3.61; protein phosphatase 6 catalytic subunit (PPP6C), with Z = +9.25 in anxiety and +11.46 in PTSD; Werner syndrome RecQ-like helicase (WRN), with Z = +3.50; and regulator of telomere elongation helicase 1 (RTEL1), with Z = +3.44. Other influential genes included cyclin-dependent kinase inhibitor 1B (CDKN1B), cyclin-dependent kinase inhibitor 2A (CDKN2A), lamin B1 (LMNB1), enhancer of zeste homolog 2 (EZH2), and suppressor of zeste 12 (SUZ12). These findings indicate that genetic risk for both conditions intersects with DNA-damage checkpoint and telomere-protection programs, consistent with broader evidence that anxiety disorders and PTSD are associated with accelerated biological aging phenotypes [4,6,7,17,18,31].

NEK4 as a novel shared senescence/checkpoint driver

NEK4 emerged as one of the strongest and most consistent gene-level signals across senescence-related sets. In the GOBP CELLULAR SENESCENCE ALL collection, NEK4 showed Z = +12.93 in anxiety and Z = +7.80 in PTSD. It represented the largest single-gene contribution to these enrichments, with 100.5% leave-one-out influence in anxiety and 21.6% in PTSD. NEK4 was also the top driver in the regulation-of-cellular-senescence set for both disorders.

NEK4 is a kinase implicated in DNA-damage response and cell-cycle checkpoint regulation. Its dominant influence in both anxiety and PTSD senescence modules suggests a shared genetic mechanism that may sensitize cells to stress-induced checkpoint activation or senescence-like states in brain tissue. Because this inference is based on genetically predicted expression, functional studies are needed before NEK4 can be considered a confirmed causal driver of stress-related brain aging.

Anxiety-enriched mitochondrial intrinsic apoptosis

Anxiety disorders showed particularly strong enrichment in intrinsic apoptosis pathways. The REACTOME INTRINSIC PATHWAY FOR APOPTOSIS ALL set produced an overall Stouffer Z of +6.43, with permutation p = 0.0056 (Table 5). Tissue-specific values were +4.94 in the nucleus accumbens, +5.33 in the caudate, and +3.48 in the hippocampus. KEGG APOPTOSIS ALL and HALLMARK APOPTOSIS ALL showed parallel positive enrichment, with overall Z values between +3.63 and +3.88.

**Table 5:** Anxiety-Enriched Intrinsic Apoptosis and Key BCL-2 / Caspase Drivers Note. For gene-level rows, the % column denotes leave-one-out influence on pathway-level Stouffer's Z-score. PTSD = posttraumatic stress disorder; anx_cc = anxiety disorder case-control phenotype; REACTOME = Reactome pathway database; KEGG = Kyoto Encyclopedia of Genes and Genomes; GOCC = Gene Ontology Cellular Component; BCL-2 = B-cell lymphoma 2; Nuc. Accumbens = nucleus accumbens; BAK1 = BCL2 antagonist/killer 1; BIK = BCL2 interacting killer; BCL2L1 = BCL2-like 1; CASP10 = caspase 10; CASP7 = caspase 7; GPX1 = glutathione peroxidase 1; n = number of genes; Z = Stouffer's combined Z-score; p = p-value.

Gene set / driver	Trait	Tissue	n	Z	p
REACTOME intrinsic pathway for apoptosis	anx_cc	Pooled	47	+6.43	.006
REACTOME intrinsic pathway for apoptosis	anx_cc	Caudate	38	+5.33	.001
REACTOME intrinsic pathway for apoptosis	anx_cc	Nuc. Accumbens	35	+4.94	< .001
REACTOME intrinsic pathway for apoptosis	anx_cc	Hippocampus	27	+3.48	.01
HALLMARK apoptosis	anx_cc	Pooled	136	+3.88	.036
KEGG apoptosis	anx_cc	Pooled	69	+3.63	-
Gene-level drivers					
BAK1--GOCC BCL-2 family complex	anx_cc	-	-	-3.92	98.90%
BIK--GOCC BCL-2 family complex	anx_cc	-	-	+2.97	65.40%
BCL2L1--GOCC BCL-2 family complex	anx_cc	-	-	+2.82	61.90%
CASP10--KEGG apoptosis	anx_cc	-	-	+6.4	20.60%
CASP7--KEGG apoptosis	ptsd	-	-	+4.84	37.30%
GPX1--HALLMARK apoptosis	ptsd	-	-	+10.78	31.80%

Key driver genes within B-cell lymphoma 2 (BCL-2) family and executioner caspase modules included BCL2 antagonist/killer 1 (BAK1), with Z = -3.92 and 98.9% leave-one-out influence; BCL2 interacting killer (BIK), with Z = +2.97; BCL2-like 1 (BCL2L1), with Z = +2.82; BCL2-associated agonist of cell death (BAD), with Z = +2.67; B-cell lymphoma 2 (BCL2), with Z = -1.18; caspase 7 (CASP7), with Z = +4.84 in PTSD but also influence in anxiety; and caspase 10 (CASP10), with Z = +6.40. Glutathione peroxidase 1 (GPX1), an antioxidant enzyme, was a major contributor in PTSD apoptosis sets, with Z = +10.78. Taken together, these results are consistent with altered mitochondrial apoptosis-threshold regulation in anxiety disorders, particularly in striatal and hippocampal circuits.

Aging-to-pruning/plasticity bridge

A bridge between aging biology and synaptic remodeling was evident across complement, microglial, structural plasticity, and presynaptic release gene sets. The complement set showed very large positive enrichment, with complement C4A (C4A) displaying Z = +11.01 in anxiety and Z = +12.33 in PTSD. Complement C1q A chain (C1QA) and complement C1q C chain (C1QC) were also influential, with C1QA showing Z = -2.84 in PTSD and C1QC showing Z = +2.47 in anxiety.

Microglial state markers suggested a possible disorder-level divergence. Triggering receptor expressed on myeloid cells 2 (TREM2) was positive in PTSD pruning contexts, with Z = +3.72, whereas triggering receptor expressed on myeloid cells 1 (TREM1) was strongly positive in anxiety, with Z = +5.54. These findings are notable given the role of TREM-family signaling in microglial biology and neurodegenerative disease contexts [32,33]. Structural plasticity genes showed consistent negative signals, including microtubule-associated protein 1A (MAP1A) with Z = -7.33 in anxiety and -10.51 in PTSD, staufen double-stranded ribonucleic acid binding protein 1 (STAU1) with Z = -7.09 and -5.91, activity-regulated cytoskeleton-associated protein (ARC) with Z = -4.64 in anxiety, and discs large membrane-associated guanylate kinase scaffold protein 4 (DLG4) with Z = -3.96 in PTSD. Presynaptic vesicle machinery was also altered. Syntaxin 1A (STX1A) reached Z = -7.01 in PTSD, while vesicle-associated membrane protein 2 (VAMP2) showed Z = +6.98 in anxiety and +3.57 in PTSD. Glutamatergic genes included glutamate metabotropic receptor 3 (GRM3), with Z = +7.36 in anxiety, and glutamate ionotropic receptor alpha-amino-3-hydroxy-5-methyl-4-isoxazolepropionic acid type subunit 1 (GRIA1), with Z = +5.35 in PTSD. These patterns suggest that aging-related stress may converge on both microglial pruning and activity-dependent synaptic remodeling, but with disorder-specific emphasis.

Supporting modules

Additional modules provided further context. Inflammatory and senescence-associated secretory phenotype-like sets, including HALLMARK INFLAMMATORY RESPONSE and HALLMARK TNFA SIGNALING VIA NFKB, showed positive enrichment, especially in anxiety, with Z values up to +4.69. Nitric oxide synthase 2 (NOS2) was a recurrent driver, with Z = +8.24 in anxiety and +7.71 in PTSD. Insulin secretion and GLP-1-related sets showed mixed but significant signals, including strong negative enrichment in PTSD for regulation of insulin secretion, with an overall Z of -6.20. Circadian clock genes showed modest positive enrichment in PTSD amygdala and nucleus accumbens, with Z values between +2.34 and +3.05. An oligodendrocyte/myelin signature was also detected, with ADULT OLIGO ALL showing Z = +5.30 in PTSD, driven largely by glycerate kinase (GLYCTK) and pleckstrin homology domain-containing B1 (PLEKHB1).

Cross-disorder contrasts and gene-level drivers

Differential testing identified several sets with statistically significant differences in magnitude between disorders (Table 6). The WANG ADIPOGENIC GENES REPRESSED BY SIRT1 ALL set was more negative in PTSD, with a mean Z difference of -1.869, Mann-Whitney U p = 0.035, and paired Cohen’s d = -0.74. HALLMARK TNFA SIGNALING VIA NFKB and REACTOME CYTOKINE SIGNALING IN IMMUNE SYSTEM showed greater effect sizes in anxiety.

**Table 6:** Cross-Disorder Differential Contrasts and Supporting Modules Note. ΔZ = mean Stouffer Z-score difference, calculated as PTSD - anx_cc; dpaired = paired Cohen's d; MWU = Mann–Whitney U test; Paired W = paired Wilcoxon test; Perm = sign-flip permutation test. Dashes indicate metrics that were not computed or did not reach the reporting threshold. PTSD = posttraumatic stress disorder; anx_cc = anxiety disorder case-control phenotype; FDR = false discovery rate; SIRT1 = sirtuin 1; TNFA = tumor necrosis factor alpha; NFKB = nuclear factor kappa B; KEGG = Kyoto Encyclopedia of Genes and Genomes; GOBP = Gene Ontology Biological Process; ADULT_OLIGO = adult oligodendrocyte gene signature; GLYCTK = glycerate kinase; PLEKHB1 = pleckstrin homology domain-containing B1.

Gene set	ΔZ	dpaired	MWU p (FDR)	Paired W p	Perm p
WANG adipogenic genes repressed by SIRT1 (ptsd < anx_cc)	-1.87	-0.74	.035	.008	.002
HALLMARK TNFA signaling via NFKB (anx_cc > ptsd)	-0.80	-0.51	.117	.044	.033
REACTOME cytokine signaling in immune system (anx_cc > ptsd)	-0.75	-0.51	-	-	-
KEGG nicotinate and nicotinamide metabolism (anx_cc > ptsd)	-1.12	-0.49	-	-	.032
GOBP cellular senescence (ptsd > anx_cc)	+0.63	+0.27	-	.024	.026
Supporting pathway-level signals					
HALLMARK inflammatory response -- anx_cc (pooled)	-	-	-	.052	.048
HALLMARK TNFA signaling via NFKB -- anx_cc (pooled, Z = +4.62)	-	-	-	.044	.047
REACTOME regulation of insulin secretion -- ptsd (pooled, Z = -6.20)	-	-	-	.079	.012
REACTOME circadian clock -- ptsd amygdala (Z = +3.05)	-	-	-	.031	-
ADULT_OLIGO -- ptsd (pooled, Z = +5.30; GLYCTK, PLEKHB1 drivers)	-	-	-	.156	.03

Across all significant sets, leave-one-out analyses identified 1,113 influential gene-disease entries. The most recurrent high-impact drivers were SIRT3 in PTSD NAD sets, NEK4 in senescence sets for both disorders, C4A in complement sets, STX1A and VAMP2 in presynaptic release sets, GRM3 in anxiety glutamatergic sets, and BAK1 and BIK in apoptosis sets. These gene-level results reinforce the pathway findings and identify specific candidates for follow-up functional studies.

## Discussion

This TWAS-based enrichment analysis suggests that genetic liability for anxiety disorders and PTSD intersects with core programs of cellular aging. Across 185 significant findings, we observed enrichment in pathways related to mitochondrial function, NAD metabolism, senescence, telomere maintenance, apoptosis, inflammation, and synaptic remodeling. Some modules showed substantial concordance between the two disorders, while others differed in direction or magnitude. These patterns may help explain why anxiety disorders and PTSD share features of accelerated biological aging but differ in clinical expression and neurocircuit involvement. The findings should be interpreted as genetically informed pathway associations, not as direct evidence of altered transcript, protein, metabolite, or cellular pathway activity in affected individuals.

A unifying model emerges from the data. Genetic risk appears to converge on a stress-aging cascade in which mitochondrial and NAD-related perturbations contribute to DNA damage and senescence checkpoint activation. These changes may then engage apoptosis and inflammatory signaling, including SASP-like responses. Downstream, these upstream cellular stress programs appear to feed into complement- and microglia-mediated synaptic pruning, structural plasticity changes, and presynaptic remodeling. This model is consistent with the broader hallmarks of the aging framework and with evidence that stress-related disorders involve bioenergetic, inflammatory, and plasticity-related disturbances [5,17,18,34,35].

Within this shared framework, PTSD showed a route marked by SIRT3 suppression and broader reduction in NAD/sirtuin tone. This may lower the threshold for SARM1-mediated axonal or synaptic disconnection. The pattern is supported by negative enrichment in NAD biosynthetic and sirtuin-related gene sets, as well as positive SARM1 signals across mitophagy and pruning collections. Work on SARM1 has shown that its activation can trigger local axon degeneration through NAD+ destruction, and reviews of programmed axon degeneration have emphasized its relevance to injury and disease [23-25,29,30]. However, the present data do not establish that SIRT3 suppression causes NAD depletion or that SARM1 is enzymatically activated in PTSD; this axis should be viewed as a testable mechanistic hypothesis. In anxiety disorders, the dominant pattern involved altered mitochondrial apoptosis thresholds, especially in striatal and hippocampal regions, together with inflammatory-redox activation involving NOS2 and tumor necrosis factor (TNF) signaling and glutamatergic/astrocytic plasticity changes involving GRM3. The most clearly shared component was the senescence, telomere, and DNA-damage module, where NEK4, ATM, NBN, POT1, TERF1, TERF2, and PPP6C emerged as recurrent drivers, and cross-disorder correlations exceeded r = 0.60.

These molecular findings map onto known neurocircuit abnormalities. Fear generalization and impaired safety learning have been linked to dysregulation in amygdala-hippocampal and amygdala-prefrontal circuits [19,20]. The pruning and plasticity signals found here, particularly those involving C4A, TREM2, TREM1, ARC, DLG4, and presynaptic vesicle machinery, could contribute to excessive or poorly timed synapse elimination in these circuits. Complement and microglial pathways are well established in developmental synapse elimination, and their reactivation or dysregulation in adult brains may contribute to disease-related circuit remodeling [26-28]. The presynaptic remodeling findings involving STX1A, VAMP2, and synaptotagmin 1 (SYT1) are also relevant because precise neurotransmitter release timing is important for extinction learning and fear memory updating [21].

The regional results also fit with known clinical patterns. Striatal involvement, especially in the caudate and nucleus accumbens, is consistent with reward-processing changes and avoidance biases in both disorders. These may be expressed differently across diagnoses, with avoidance more prominent in anxiety disorders and intrusive memory, hyperarousal, and trauma-related reactivity more characteristic of PTSD [11,12]. The mitochondrial and NAD-related signals also align with reports of bioenergetic dysfunction and oxidative stress in stress-related conditions [35,36]. Energetic insufficiency at synapses may make them more vulnerable to functional disconnection or elimination, particularly under chronic stress.

From a translational standpoint, the findings support the development of biologically informed subtypes rather than treating anxiety disorders and PTSD as single, uniform categories. At this stage, these subtype labels are intended as research constructs for biomarker testing and trial enrichment, not as validated diagnostic categories. A mitochondrial-resilience PTSD subtype could be defined by low NAD/sirtuin tone, elevated oxidative stress markers, and evidence of axonal or synaptic injury, such as neurofilament light chain. An axon-vulnerability PTSD subtype might be further characterized by combined NAD/SARM1 signatures and diffusion magnetic resonance imaging (MRI) or connectivity abnormalities. For anxiety disorders, a mitochondrial-apoptotic subtype could be marked by elevated apoptosis-threshold gene signals together with redox and inflammatory markers. An inflammatory-redox subtype might be identified by prominent NOS2/TNF and SASP-like profiles. A broader accelerated-aging stress disorder subtype, spanning both diagnoses, could be defined by epigenetic age acceleration, telomere length, and senescence-associated inflammatory markers. Such stratification would allow future trials to enrich for biologically relevant subgroups and better match patients to existing or emerging interventions.

Several biomarker panels follow directly from the top modules. A PTSD mitochondrial-resilience panel could combine NAD metabolomics, including oxidized/reduced nicotinamide adenine dinucleotide (NAD+/NADH) ratio, nicotinamide (NAM), and nicotinamide mononucleotide (NMN), with SIRT3-informed polygenic or predicted-expression scores, redox markers such as reduced/oxidized glutathione (GSH/GSSG) and 8-hydroxy-2′-deoxyguanosine (8-OHdG), and synaptic injury indicators such as neurofilament light. An aging panel applicable to both disorders could include GrimAge or DunedinPACE epigenetic clocks, leukocyte telomere length, p16/cyclin-dependent kinase inhibitor 2A (p16/CDKN2A) expression, and SASP cytokines. Pruning and plasticity signatures could be assessed through complement components such as C4A, microglial state markers such as the TREM2/TREM1 ratio, and synaptic proteins measured in neuron-derived extracellular vesicles. These panels could be tested against symptom clusters, treatment resistance, cognitive impairment, and longitudinal outcomes in existing cohorts.

The same model suggests several therapeutic hypotheses. Biomarker-guided aerobic exercise and sleep stabilization are immediately testable, low-risk interventions that engage mitochondrial biogenesis, NAD metabolism, and plasticity processes. Metabolic optimization, including GLP-1-related pathways where relevant, may be useful in subgroups showing insulin secretion or glucose-homeostasis signals. NAD-precursor or sirtuin-modulating strategies could be evaluated in enriched PTSD mitochondrial-resilience subgroups, while SARM1-pathway modulators remain a longer-term possibility pending further validation. Plasticity-focused interventions could include optimized sleep timing around exposure sessions, exercise augmentation of trauma-focused therapy, and circadian-aligned treatment scheduling in patients with high pruning or plasticity gene signatures. Glutamate-modulating or anti-inflammatory adjuncts may be relevant for GRM3-high or NOS2-high anxiety subgroups.

The study has several strengths. Brain-region-specific TWAS weights allowed more tissue-relevant interpretation than locus-level GWAS alone. The multi-gene-set design made it possible to detect coordinated pathway signals rather than isolated gene effects. Cross-disorder comparisons identified both shared and disorder-specific biology, and leave-one-out analyses provided gene-level resolution. Integrating aging modules with synaptic plasticity and pruning sets also provided a mechanistic bridge that has often been missing from prior genetic studies of anxiety disorders and PTSD.

Several limitations should also be noted. TWAS estimates genetically predicted expression and does not directly measure transcript or protein levels in patient tissue, so causal inference remains provisional. Some of the strongest pathway signals were strongly influenced by individual genes, including SIRT3, NEK4, and C4A. This indicates that pathway results can be sensitive to dominant drivers and highlights the need for colocalization, replication, and functional validation. Complement and human leukocyte antigen (HLA)-related findings require caution because of extended linkage disequilibrium and possible immune-cell composition effects. Major histocompatibility complex (MHC)-exclusion analyses were performed, but they cannot fully remove this concern. The analysis also depends on curated gene-set definitions, which vary by database and often contain overlapping genes; therefore, related pathway hits should not be interpreted as statistically independent biological processes. Both GWAS inputs were restricted to European-ancestry participants, which improves ancestry consistency but limits generalizability to other populations. No pathway family survived the most stringent family-wise FDR summary, so the reported findings should be considered hypothesis-generating and prioritized for replication. Finally, the cross-sectional nature of the GWAS data prevents definitive conclusions about temporal order or causality in the proposed cascade.

Future work should prioritize functional validation in human-induced pluripotent stem cell-derived neurons, astrocytes, and microglia. Targeted perturbation of SIRT3, SARM1, NEK4, and key pruning genes under glucocorticoid or inflammatory stress would be especially informative. Clinical cohort studies should combine the proposed biomarker panels with longitudinal symptom tracking, extinction-learning tasks, and multimodal neuroimaging, including diffusion MRI, resting-state connectivity, and fear-conditioning functional magnetic resonance imaging (fMRI). Subtype-enriched randomized trials comparing standard care with adjunctive mitochondrial support or plasticity-enhancing interventions would be a logical step toward precision treatment.

In sum, the data support an association-based model in which genetic risk for anxiety disorders and PTSD converges on aging biology at the synapse while diverging in the molecular route taken. This framework moves beyond broad ideas of stress or inflammation and toward mechanistically grounded candidate subtypes that may improve risk stratification, biomarker development, and targeted intervention strategies.

## Conclusions

This TWAS enrichment study suggests that genetic liability for anxiety disorders and PTSD intersects with core programs of cellular aging in brain tissue. The findings support a hypothesis-generating stress-aging-synapse cascade that is shared across disorders but also contains disorder-specific branches. PTSD risk showed a pronounced signature of NAD/sirtuin suppression and SARM1-linked synaptic vulnerability, whereas anxiety risk emphasized mitochondrial apoptosis thresholds and inflammatory-glutamatergic plasticity changes. Both disorders shared senescence, telomere, and DNA-damage programs.

These findings support the hypothesis of biologically distinct subtypes, including a mitochondrial-resilience PTSD subtype and a plasticity/pruning profile that may help predict exposure-therapy response. They also point to biomarker panels and adjunctive treatment strategies that could be tested in future studies. By connecting molecular aging mechanisms to circuit-level dysfunction, this work provides a foundation for moving from descriptive genetic associations toward more precise approaches to risk assessment and treatment in anxiety disorders and PTSD.

## Appendices

Supplementary profile-proximity analyses, per-disease enrichment results, differential testing, concordance metrics, and gene-level leave-one-out influence analyses are provided in Tables 7-12.

**Table 7:** Profile Proximity: Pearson and Spearman Correlations Note. r = Pearson correlation; ρ = Spearman rank correlation; cos = cosine similarity; ANX = anx_cc; p values reported as in source (0 indicates p < 1 × 10⁻⁶).

#	Gene Set	Comparison	n	r	r p	ρ	ρ p	cos
1	GOBP_CELLULAR_SENESCENCE_ALL	PTSD vs ANX	75	0.599	0	0.537	1.00E-06	0.574
2	GOBP_INTRINSIC_APOPTOTIC_SIGNALING_PATHWAY_ALL	PTSD vs ANX	264	0.465	0	0.435	0	0.467
3	GOBP_REGULATION_OF_CELLULAR_SENESCENCE_ALL	PTSD vs ANX	35	0.757	0	0.625	5.90E-05	0.76
4	GOBP_REGULATION_OF_SYNAPTIC_PLASTICITY_ALL	PTSD vs ANX	151	0.596	0	0.512	0	0.591
5	HALLMARK_APOPTOSIS_ALL	PTSD vs ANX	131	0.514	0	0.524	0	0.523
6	HALLMARK_OXIDATIVE_PHOSPHORYLATION_ALL	PTSD vs ANX	149	0.451	0	0.397	1.00E-06	0.451
7	MOOTHA_MITOCHONDRIA_ALL	PTSD vs ANX	353	0.504	0	0.395	0	0.505
8	MOOTHA_PGC_ALL	PTSD vs ANX	327	0.479	0	0.402	0	0.479
9	REACTOME_TELOMERE_ALL	PTSD vs ANX	54	0.702	0	0.596	2.00E-06	0.703
10	REACTOME_CELLULAR_SENESCENCE_ALL	PTSD vs ANX	100	0.464	1.00E-06	0.364	0.000201	0.454
11	REACTOME_SENESCENCE_ALL	PTSD vs ANX	100	0.464	1.00E-06	0.364	0.000201	0.454
12	SAUL_SEN_MAYO_ALL	PTSD vs ANX	91	0.488	1.00E-06	0.436	1.60E-05	0.48
13	HLA_COMPLEX_ALL	PTSD vs ANX	16	0.885	5.00E-06	0.868	1.30E-05	0.886
14	ADULT_OLIGO_ALL	PTSD vs ANX	25	0.735	2.90E-05	0.484	0.01426	0.752
15	REACTOME_REGULATION_OF_INSULIN_SECRETION_ALL	PTSD vs ANX	58	0.501	6.10E-05	0.4	0.001876	0.522
16	HALLMARK_TNFA_SIGNALING_VIA_NFKB	PTSD vs ANX	20	0.744	0.00017	0.34	0.1426	0.714
17	HALLMARK_PI3K_AKT_MTOR_SIGNALING_ALL	PTSD vs ANX	87	0.386	0.000227	0.34	0.001277	0.379
18	KEGG_LTP_ALL	PTSD vs ANX	52	0.481	0.000304	0.421	0.001889	0.489
19	HALLMARK_INFLAMMATORY_RESPONSE	PTSD vs ANX	21	0.67	0.000882	0.323	0.1527	0.646
20	GOBP_INSULIN_SECRETION_INVOLVED_IN_CELLULAR_RESPONSE_TO_GLUCOSE_STIMULUS_ALL	PTSD vs ANX	53	0.441	0.00094	0.444	0.000874	0.485
21	REACTOME_SYNTHESIS_SECRETION_AND_INACTIVATION_OF_GLP_1_ALL	PTSD vs ANX	12	0.813	0.001307	0.545	0.06661	0.81
22	KEGG_APOPTOSIS_ALL	PTSD vs ANX	68	0.38	0.001403	0.411	0.000493	0.389
23	REACTOME_MITOPHAGY	PTSD vs ANX	19	0.632	0.003662	0.386	0.1027	0.625
24	MONOAMINES_ALL	PTSD vs ANX	69	0.342	0.003975	0.389	0.000947	0.342
25	ADULT_ASTRO_ALL	PTSD vs ANX	34	0.47	0.005031	0.432	0.01062	0.476
26	REACTOME_COMPLEMENT_ALL	PTSD vs ANX	34	0.468	0.005214	0.082	0.6446	0.486
27	REACTOME_ACTIVATION_OF_INNATE_IMMUNE_RESPONSE	PTSD vs ANX	15	0.674	0.005886	0.536	0.03957	0.674
28	GOCC_BCL_2_FAMILY_PROTEIN_COMPLEX_ALL	PTSD vs ANX	10	0.795	0.006	0.855	0.001637	0.783
29	GOBP_GLUTAMATERGIC_ALL	PTSD vs ANX	75	0.299	0.009043	0.319	0.005283	0.302
30	KEGG_DOPAMINERGIC_SYNAPSE	PTSD vs ANX	16	0.627	0.009272	0.597	0.01461	0.601
31	REACTOME_NEUROTRANSMITTER_RELEASE_CYCLE	PTSD vs ANX	11	0.697	0.01725	0.727	0.0112	0.691

**Table 8:** Per-Disease Enrichment Results Note. ACC BA24 = Anterior Cingulate Cortex BA24; Aggregate = cross-tissue aggregate; - = not available. Tissues are S-PrediXcan brain regions.

#	Gene Set	Disease	Tissue	n	Stouffer Z	Perm p	Wilcoxon p
1	REACTOME_INTRINSIC_PATHWAY_FOR_APOPTOSIS_ALL	anx_cc	Nucleus Accumbens	35	4.936	-	1.92E-04
2	REACTOME_INTRINSIC_PATHWAY_FOR_APOPTOSIS_ALL	anx_cc	Caudate	38	5.334	-	0.000979
3	WANG_ADIPOGENIC_GENES_REPRESSED_BY_SIRT1_ALL	ptsd	Caudate	12	-3.482	-	4.88E-03
4	REACTOME_INTRINSIC_PATHWAY_FOR_APOPTOSIS_ALL	ptsd	Caudate	38	3.345	-	0.005443
5	REACTOME_INTRINSIC_PATHWAY_FOR_APOPTOSIS_ALL	anx_cc	Aggregate	47	6.431	0.005599	0.03095
6	REACTOME_INTRINSIC_PATHWAY_FOR_APOPTOSIS_ALL	anx_cc	Hippocampus	27	3.475	-	1.04E-02
7	REACTOME_REGULATION_OF_INSULIN_SECRETION_ALL	ptsd	Aggregate	58	-6.199	0.0115	0.07949
8	REACTOME_NICOTINATE_METABOLISM_ALL	anx_cc	Hippocampus	22	2.717	-	0.01273
9	WP_NAD_BIOSYNTHETIC_PATHWAYS_ALL	ptsd	Aggregate	19	-5.877	0.015	9.55E-02
10	KEGG_APOPTOSIS_ALL	anx_cc	Caudate	50	3.04E+00	-	0.01574
11	HALLMARK_PI3K_AKT_MTOR_SIGNALING_ALL	ptsd	Amygdala	46	3.05E+00	-	0.02014
12	WP_NAD_BIOSYNTHETIC_PATHWAYS_ALL	ptsd	ACC BA24	17	-2.962	-	2.02E-02
13	KEGG_NICOTINATE_AND_NICOTINAMIDE_METABOLISM_ALL	anx_cc	Caudate	16	2.88E+00	-	2.14E-02
14	GOBP_INSULIN_SECRETION_INVOLVED_IN_CELLULAR_RESPONSE_TO_GLUCOSE_STIMULUS_ALL	anx_cc	Amygdala	32	3.26E+00	-	0.02279
15	REACTOME_CYTOKINE_SIGNALING_IN_IMMUNE_SYSTEM	ptsd	Caudate	11	2.18E+00	-	0.02441
16	WP_NAD_BIOSYNTHETIC_PATHWAYS_ALL	ptsd	Amygdala	14	-2.954	-	0.02454
17	GOBP_INSULIN_SECRETION_INVOLVED_IN_CELLULAR_RESPONSE_TO_GLUCOSE_STIMULUS_ALL	ptsd	Amygdala	31	3.933	-	0.02465
18	REACTOME_TELOMERE_ALL	anx_cc	Caudate	44	2.922	-	0.02517
19	GOBP_INSULIN_SECRETION_INVOLVED_IN_CELLULAR_RESPONSE_TO_GLUCOSE_STIMULUS_ALL	anx_cc	Caudate	35	3.768	-	0.0263
20	ADULT_OLIGO_ALL	ptsd	Hippocampus	17	3.757	-	0.02667
21	GOBP_INSULIN_SECRETION_INVOLVED_IN_CELLULAR_RESPONSE_TO_GLUCOSE_STIMULUS_ALL	anx_cc	Aggregate	54	5.259	0.028	0.05699
22	ADULT_OLIGO_ALL	ptsd	Aggregate	25	5.3	0.0299	0.1563
23	REACTOME_CIRCADIAN_CLOCK	ptsd	Amygdala	6	3.052	-	0.03125
24	REACTOME_CIRCADIAN_CLOCK	ptsd	Nucleus Accumbens	6	2.338	-	0.03125
25	HALLMARK_INFLAMMATORY_RESPONSE	anx_cc	ACC BA24	11	2.994	-	0.03223
26	GOBP_INSULIN_SECRETION_INVOLVED_IN_CELLULAR_RESPONSE_TO_GLUCOSE_STIMULUS_ALL	ptsd	Nucleus Accumbens	35	2.687	-	0.03271
27	REACTOME_REGULATION_OF_INSULIN_SECRETION_ALL	ptsd	Frontal Cortex BA9	40	-3.745	-	0.0344
28	GOBP_GLUTAMATERGIC_ALL	anx_cc	Frontal Cortex BA9	54	1.661	-	0.03453
29	GOBP_CELLULAR_SENESCENCE_ALL	ptsd	Amygdala	53	2.686	-	0.0379
30	REACTOME_CYTOKINE_SIGNALING_IN_IMMUNE_SYSTEM	anx_cc	Aggregate	17	4.946	0.0389	0.004639
31	HALLMARK_APOPTOSIS_ALL	anx_cc	Hippocampus	80	1.926	-	0.04059
32	KEGG_LTP_ALL	ptsd	Amygdala	27	2.258	-	0.04098
33	KEGG_APOPTOSIS_ALL	ptsd	Frontal Cortex BA9	48	2.564	-	0.041
34	WANG_ADIPOGENIC_GENES_REPRESSED_BY_SIRT1_ALL	ptsd	Aggregate	18	-4.984	0.0411	0.08143
35	KEGG_DOPAMINERGIC_SYNAPSE	ptsd	Caudate	12	-3.869	-	0.04248
36	GOBP_INSULIN_SECRETION_INVOLVED_IN_CELLULAR_RESPONSE_TO_GLUCOSE_STIMULUS_ALL	anx_cc	Frontal Cortex BA9	40	2.995	-	0.04458
37	REACTOME_CELLULAR_SENESCENCE_ALL	anx_cc	Amygdala	66	-2.393	-	0.0459
38	REACTOME_SENESCENCE_ALL	anx_cc	Amygdala	66	-2.393	-	0.0459
39	WANG_ADIPOGENIC_GENES_REPRESSED_BY_SIRT1_ALL	ptsd	Nucleus Accumbens	7	-3.631	-	0.04688
40	HALLMARK_TNFA_SIGNALING_VIA_NFKB	anx_cc	Aggregate	20	4.619	0.04739	0.04421
41	HALLMARK_INFLAMMATORY_RESPONSE	anx_cc	Aggregate	21	4.693	0.04779	0.05222
42	WP_NAD_BIOSYNTHETIC_PATHWAYS_ALL	ptsd	Hippocampus	13	-3.416	-	0.04785
43	HALLMARK_TNFA_SIGNALING_VIA_NFKB	anx_cc	Amygdala	11	2.633	-	0.04883
44	HALLMARK_OXIDATIVE_PHOSPHORYLATION_ALL	anx_cc	Amygdala	105	2.82	-	0.0506
45	WP_GLP1_FROM_INTESTINE_AND_PANCREAS_AND_ROLE_IN_GLUCOSE_HOMEOSTASIS_ALL	anx_cc	Aggregate	4	4.618	0.0514	-
46	GOBP_REGULATION_OF_SYNAPTIC_PLASTICITY_ALL	anx_cc	Caudate	96	2.093	-	0.05233
47	REACTOME_CYTOKINE_SIGNALING_IN_IMMUNE_SYSTEM	anx_cc	Caudate	11	2.51	-	0.05371
48	GOBP_CELLULAR_SENESCENCE_ALL	ptsd	Hippocampus	52	2.924	-	0.05466
49	GOBP_INSULIN_SECRETION_INVOLVED_IN_CELLULAR_RESPONSE_TO_GLUCOSE_STIMULUS_ALL	ptsd	Caudate	35	3.21	-	0.05787
50	MONOAMINES_ALL	anx_cc	Frontal Cortex BA9	46	2.111	-	0.05826
51	GOBP_INSULIN_SECRETION_INVOLVED_IN_CELLULAR_RESPONSE_TO_GLUCOSE_STIMULUS_ALL	ptsd	Aggregate	54	4.519	0.06159	0.1141
52	KEGG_MEDICUS_REFERENCE_EP_NE_ADRB_CAMP_SIGNALING_PATHWAY_ALL	ptsd	ACC BA24	6	3.195	-	0.0625
53	MONOAMINES_ALL	anx_cc	Hippocampus	40	2.175	-	0.06415
54	REACTOME_INTRINSIC_PATHWAY_FOR_APOPTOSIS_ALL	ptsd	Nucleus Accumbens	36	2.252	-	0.0668
55	KEGG_DOPAMINERGIC_SYNAPSE	anx_cc	Hippocampus	11	3.421	-	0.06738
56	REACTOME_MITOPHAGY	anx_cc	ACC BA24	13	2.46	-	0.06811
57	HALLMARK_APOPTOSIS_ALL	anx_cc	ACC BA24	84	2.271	-	0.06882
58	GOBP_REGULATION_OF_SYNAPTIC_PLASTICITY_ALL	anx_cc	Hippocampus	99	2.248	-	0.06936
59	MONOAMINES_ALL	anx_cc	Amygdala	41	2.378	-	0.07141
60	GOCC_BCL_2_FAMILY_PROTEIN_COMPLEX_ALL	anx_cc	Nucleus Accumbens	9	2.491	-	0.07422
61	HALLMARK_TNFA_SIGNALING_VIA_NFKB	anx_cc	Hippocampus	9	2.752	-	0.07422
62	KEGG_NICOTINATE_AND_NICOTINAMIDE_METABOLISM_ALL	anx_cc	Aggregate	22	4.193	0.07589	0.03012
63	GOBP_SYNAPSE_PRUNING_ALL	ptsd	Nucleus Accumbens	12	2.132	-	0.07715
64	ADULT_OLIGO_ALL	anx_cc	Aggregate	25	4.111	0.07779	0.4418
65	REACTOME_CYTOKINE_SIGNALING_IN_IMMUNE_SYSTEM	anx_cc	Hippocampus	8	2.286	-	0.07812
66	WP_NAD_METABOLISM_SIRTUINS_AND_AGING_ALL	ptsd	Frontal Cortex BA9	7	-2.103	-	0.07812
67	REACTOME_ENERGY_DEPENDENT_REGULATION_OF_MTOR_BY_LKB1_AMPK_ALL	ptsd	ACC BA24	19	-1.970	-	0.07988
68	HALLMARK_TNFA_SIGNALING_VIA_NFKB	anx_cc	Nucleus Accumbens	13	2.574	-	0.08032
69	HLA_COMPLEX_ALL	anx_cc	Aggregate	16	-4.070	0.08469	0.5282
70	REACTOME_REGULATION_OF_INSULIN_SECRETION_ALL	ptsd	Amygdala	37	-2.753	-	0.09129
71	KEGG_NICOTINATE_AND_NICOTINAMIDE_METABOLISM_ALL	anx_cc	Amygdala	13	2.216	-	0.09424
72	WP_NAD_BIOSYNTHETIC_PATHWAYS_ALL	ptsd	Nucleus Accumbens	15	-2.500	-	0.0946
73	REACTOME_REGULATION_OF_INSULIN_SECRETION_ALL	ptsd	Caudate	43	-3.060	-	0.09469
74	HALLMARK_APOPTOSIS_ALL	anx_cc	Aggregate	136	3.88	0.09539	0.03565
75	GOBP_CELLULAR_SENESCENCE_ALL	ptsd	Aggregate	76	4.038	0.09789	0.1457
76	REACTOME_NICOTINATE_METABOLISM_ALL	ptsd	Amygdala	17	-2.074	-	0.09837
77	MONOAMINES_ALL	anx_cc	ACC BA24	40	2.482	-	0.1029
78	WANG_ADIPOGENIC_GENES_REPRESSED_BY_SIRT1_ALL	ptsd	ACC BA24	14	-2.161	-	0.104
79	ADULT_OLIGO_ALL	ptsd	Nucleus Accumbens	15	4.113	-	0.107
80	GOBP_INSULIN_SECRETION_INVOLVED_IN_CELLULAR_RESPONSE_TO_GLUCOSE_STIMULUS_ALL	anx_cc	Nucleus Accumbens	36	2.307	-	0.1079
81	REACTOME_CYTOKINE_SIGNALING_IN_IMMUNE_SYSTEM	anx_cc	Nucleus Accumbens	8	2.762	-	0.1094
82	ADULT_ASTRO_ALL	ptsd	Amygdala	26	-2.271	-	0.1105
83	REACTOME_REGULATION_OF_INSULIN_SECRETION_ALL	anx_cc	Amygdala	36	-2.187	-	0.1115
84	REACTOME_CELLULAR_SENESCENCE_ALL	anx_cc	Aggregate	104	-3.647	0.1146	0.03959
85	REACTOME_SENESCENCE_ALL	anx_cc	Aggregate	104	-3.647	0.1146	0.03959
86	HALLMARK_OXIDATIVE_PHOSPHORYLATION_ALL	anx_cc	Caudate	127	2.521	-	0.1172
87	KEGG_APOPTOSIS_ALL	anx_cc	ACC BA24	42	1.96	-	0.1218
88	KEGG_APOPTOSIS_ALL	anx_cc	Aggregate	69	3.632	0.1235	0.2217
89	MOOTHA_MITOCHONDRIA_ALL	anx_cc	Caudate	283	2.169	-	0.127
90	REACTOME_REGULATION_OF_INSULIN_SECRETION_ALL	ptsd	ACC BA24	41	-2.897	-	0.1272
91	HLA_COMPLEX_ALL	ptsd	Aggregate	17	-3.717	0.1283	0.7819
92	GOBP_CELLULAR_SENESCENCE_ALL	anx_cc	Frontal Cortex BA9	53	-2.049	-	0.129
93	WP_NAD_BIOSYNTHETIC_PATHWAYS_ALL	ptsd	Caudate	12	-2.969	-	0.1294
94	GOBP_INTRINSIC_APOPTOTIC_SIGNALING_PATHWAY_ALL	ptsd	Caudate	193	2.517	-	0.1295
95	GOBP_GLUTAMATERGIC_ALL	ptsd	Nucleus Accumbens	52	1.981	-	0.1306
96	REACTOME_GLUCAGON_LIKE_PEPTIDE_1_GLP1_REGULATES_INSULIN_SECRETION_ALL	ptsd	Amygdala	17	-2.072	-	0.1324
97	REACTOME_REGULATION_OF_INSULIN_SECRETION_ALL	ptsd	Hippocampus	37	-2.783	-	0.1353
98	REACTOME_INTRINSIC_PATHWAY_FOR_APOPTOSIS_ALL	anx_cc	Amygdala	26	2.069	-	0.1358
99	REACTOME_CELLULAR_SENESCENCE_ALL	anx_cc	Frontal Cortex BA9	69	-2.054	-	0.1376
100	REACTOME_SENESCENCE_ALL	anx_cc	Frontal Cortex BA9	69	-2.054	-	0.1376
101	REACTOME_ENERGY_DEPENDENT_REGULATION_OF_MTOR_BY_LKB1_AMPK_ALL	anx_cc	Hippocampus	18	2.384	-	0.1415
102	REACTOME_TELOMERE_ALL	ptsd	Hippocampus	40	2.797	-	0.1423
103	GOBP_INTRINSIC_APOPTOTIC_SIGNALING_PATHWAY_ALL	anx_cc	Nucleus Accumbens	190	2.461	-	0.1427
104	REACTOME_ENERGY_DEPENDENT_REGULATION_OF_MTOR_BY_LKB1_AMPK_ALL	anx_cc	ACC BA24	19	-2.450	-	0.1447
105	GOBP_INTRINSIC_APOPTOTIC_SIGNALING_PATHWAY_ALL	anx_cc	Caudate	193	2.44	-	0.1456
106	KEGG_APOPTOSIS_ALL	anx_cc	Hippocampus	40	1.977	-	0.1461
107	REACTOME_REGULATION_OF_INSULIN_SECRETION_ALL	anx_cc	Caudate	44	-1.984	-	0.1542
108	GOBP_REGULATION_OF_CELLULAR_SENESCENCE_ALL	ptsd	Hippocampus	19	2.24	-	0.1564
109	KEGG_NICOTINATE_AND_NICOTINAMIDE_METABOLISM_ALL	anx_cc	Frontal Cortex BA9	17	2.377	-	0.1594
110	KEGG_NICOTINATE_AND_NICOTINAMIDE_METABOLISM_ALL	anx_cc	Nucleus Accumbens	17	2.254	-	0.1594
111	ADULT_ASTRO_ALL	ptsd	Aggregate	34	-3.341	0.1594	0.1207
112	GOBP_POSITIVE_REGULATION_OF_MICROGLIAL_ACTIVATION	ptsd	Caudate	10	2.146	-	0.1602
113	HLA_COMPLEX_ALL	anx_cc	Amygdala	10	-2.997	-	0.1602
114	HALLMARK_OXIDATIVE_PHOSPHORYLATION_ALL	anx_cc	Nucleus Accumbens	116	2.096	-	0.1608
115	GOBP_CELLULAR_SENESCENCE_ALL	ptsd	Caudate	52	2.182	-	0.1663
116	REACTOME_NICOTINATE_METABOLISM_ALL	anx_cc	Aggregate	27	3.227	0.1674	0.1482
117	REACTOME_REGULATION_OF_INSULIN_SECRETION_ALL	anx_cc	Aggregate	61	-3.179	0.1717	0.2101
118	GOBP_INSULIN_SECRETION_INVOLVED_IN_CELLULAR_RESPONSE_TO_GLUCOSE_STIMULUS_ALL	ptsd	Frontal Cortex BA9	38	2.139	-	0.1769
119	GOCC_BCL_2_FAMILY_PROTEIN_COMPLEX_ALL	ptsd	Aggregate	10	3.224	0.178	0.1289
120	GOBP_INTRINSIC_APOPTOTIC_SIGNALING_PATHWAY_ALL	anx_cc	Aggregate	270	3.155	0.1832	0.4022
121	MONOAMINES_ALL	anx_cc	Aggregate	70	3.092	0.1836	0.1165
122	GOBP_INSULIN_SECRETION_INVOLVED_IN_CELLULAR_RESPONSE_TO_GLUCOSE_STIMULUS_ALL	ptsd	Hippocampus	37	2.786	-	0.1866
123	HALLMARK_APOPTOSIS_ALL	ptsd	Nucleus Accumbens	88	2.045	-	0.1872
124	SAUL_SEN_MAYO_ALL	ptsd	Caudate	60	-1.999	-	0.1901
125	HLA_COMPLEX_ALL	anx_cc	Hippocampus	10	-2.701	-	0.1934
126	REACTOME_MITOPHAGY	anx_cc	Caudate	14	2.194	-	0.1937
127	REACTOME_INTRINSIC_PATHWAY_FOR_APOPTOSIS_ALL	ptsd	Aggregate	45	3.087	0.1974	0.1099
128	REACTOME_TELOMERE_ALL	anx_cc	Aggregate	55	2.967	0.2005	0.2166
129	WANG_ADIPOGENIC_GENES_REPRESSED_BY_SIRT1_ALL	ptsd	Amygdala	9	-2.784	-	0.2031
130	HALLMARK_INFLAMMATORY_RESPONSE	anx_cc	Nucleus Accumbens	12	2.008	-	0.2036
131	KEGG_LTP_ALL	ptsd	Nucleus Accumbens	29	2.166	-	0.2053
132	REACTOME_COMPLEMENT_ALL	anx_cc	Aggregate	40	2.964	0.207	0.2301
133	GOMF_NADPLUS_BINDING_ALL	ptsd	Caudate	15	-2.095	-	0.2078
134	GOBP_CAMKK_AMPK_SIGNALING_CASCADE_ALL	anx_cc	Aggregate	10	-2.867	0.2103	0.04883
135	ADULT_OLIGO_ALL	anx_cc	Nucleus Accumbens	16	3.016	-	0.2114
136	REACTOME_CIRCADIAN_CLOCK	ptsd	Aggregate	9	2.963	0.2119	0.4258
137	REACTOME_NEUROTRANSMITTER_RELEASE_CYCLE	ptsd	Amygdala	6	1.972	-	0.2188
138	GOBP_NAD_TRANSPORT_ALL	anx_cc	Aggregate	3	2.743	0.2216	-
139	MOOTHA_MITOCHONDRIA_ALL	anx_cc	Aggregate	360	2.938	0.2219	0.4392
140	REACTOME_MITOPHAGY	ptsd	Hippocampus	17	-1.962	-	0.2247
141	HALLMARK_OXIDATIVE_PHOSPHORYLATION_ALL	anx_cc	Aggregate	153	2.77	0.2321	0.6483
142	HALLMARK_APOPTOSIS_ALL	ptsd	Aggregate	131	2.941	0.2347	0.2856
143	KEGG_LTP_ALL	anx_cc	Nucleus Accumbens	32	2.062	-	0.2462
144	HLA_COMPLEX_ALL	ptsd	ACC BA24	9	-3.282	-	0.25
145	GOBP_POSITIVE_REGULATION_OF_MICROGLIAL_ACTIVATION	ptsd	Aggregate	16	2.65	0.2664	0.3225
146	GOBP_NAD_TRANSPORT_ALL	ptsd	Aggregate	3	2.511	0.2724	-
147	REACTOME_COMPLEMENT_ALL	ptsd	Aggregate	35	2.625	0.2738	0.9523
148	SAUL_SEN_MAYO_ALL	ptsd	Aggregate	92	-2.664	0.2747	0.3422
149	REACTOME_MITOPHAGY	anx_cc	Aggregate	19	2.507	0.2759	0.2413
150	GOMF_NADPLUS_BINDING_ALL	ptsd	Aggregate	17	-2.493	0.2969	0.5171
151	HLA_COMPLEX_ALL	anx_cc	ACC BA24	9	-1.990	-	0.3008
152	REACTOME_ENERGY_DEPENDENT_REGULATION_OF_MTOR_BY_LKB1_AMPK_ALL	ptsd	Aggregate	25	-2.438	0.3069	0.1014
153	KEGG_LTP_ALL	anx_cc	Aggregate	56	2.387	0.3098	0.5036
154	KEGG_MEDICUS_REFERENCE_EP_NE_ADRB_CAMP_SIGNALING_PATHWAY_ALL	ptsd	Aggregate	12	2.361	0.3195	0.3804
155	WP_NAD_METABOLISM_ALL	anx_cc	Aggregate	14	2.277	0.3204	0.1937
156	HALLMARK_INFLAMMATORY_RESPONSE	anx_cc	Hippocampus	10	1.994	-	0.3223
157	GOBP_SYNAPSE_PRUNING_ALL	ptsd	Aggregate	17	2.365	0.323	0.2069
158	ADULT_OLIGO_ALL	anx_cc	Hippocampus	18	2.34	-	0.3247
159	GOBP_REGULATION_OF_CELLULAR_SENESCENCE_ALL	ptsd	Aggregate	35	2.33	0.3292	0.4036
160	ADULT_OLIGO_ALL	ptsd	Caudate	15	2.698	-	0.3303
161	GOBP_REGULATION_OF_SYNAPTIC_PLASTICITY_ALL	ptsd	Nucleus Accumbens	86	-2.356	-	0.3348
162	REACTOME_REGULATION_OF_INSULIN_SECRETION_ALL	ptsd	Nucleus Accumbens	36	-1.983	-	0.3382
163	GOMF_NADPLUS_BINDING_ALL	anx_cc	Aggregate	17	2.191	0.3475	0.1594
164	GOBP_REGULATION_OF_CELLULAR_SENESCENCE_ALL	anx_cc	Aggregate	36	2.203	0.348	0.6028
165	WP_NAD_METABOLISM_SIRTUINS_AND_AGING_ALL	ptsd	Aggregate	10	-2.207	0.3489	1
166	KEGG_LTP_ALL	ptsd	Aggregate	52	2.291	0.3495	0.3344
167	HLA_COMPLEX_ALL	anx_cc	Nucleus Accumbens	13	-2.175	-	0.3757
168	HLA_COMPLEX_ALL	ptsd	Nucleus Accumbens	13	-2.620	-	0.4143
169	GOBP_INTRINSIC_APOPTOTIC_SIGNALING_PATHWAY_ALL	ptsd	Aggregate	266	2.001	0.4226	0.9692
170	HLA_COMPLEX_ALL	anx_cc	Caudate	12	-2.188	-	0.4238
171	HLA_COMPLEX_ALL	ptsd	Hippocampus	10	-2.438	-	0.4316
172	REACTOME_COMPLEMENT_ALL	ptsd	Hippocampus	22	2	-	0.5028
173	HLA_COMPLEX_ALL	ptsd	Frontal Cortex BA9	11	-2.198	-	0.5195
174	REACTOME_CIRCADIAN_CLOCK	ptsd	Frontal Cortex BA9	6	2.252	-	0.5625
175	REACTOME_COMPLEMENT_ALL	ptsd	ACC BA24	19	2.009	-	0.7086
176	ADULT_OLIGO_ALL	anx_cc	ACC BA24	12	2.014	-	0.8501
177	GOBP_NAD_TRANSPORT_ALL	anx_cc	Caudate	3	2.374	-	-
178	GOBP_NAD_TRANSPORT_ALL	anx_cc	Frontal Cortex BA9	3	2.062	-	-
179	GOCC_BCL_2_FAMILY_PROTEIN_COMPLEX_ALL	ptsd	Hippocampus	4	2.314	-	-
180	REACTOME_NEUROTRANSMITTER_RELEASE_CYCLE	anx_cc	Amygdala	5	2.844	-	-
181	REACTOME_SIRT1_NEGATIVELY_REGULATES_RRNA_EXPRESSION_ALL	ptsd	ACC BA24	5	-2.015	-	-
182	REACTOME_SIRT1_NEGATIVELY_REGULATES_RRNA_EXPRESSION_ALL	ptsd	Hippocampus	4	-2.120	-	-
183	WP_GLP1_FROM_INTESTINE_AND_PANCREAS_AND_ROLE_IN_GLUCOSE_HOMEOSTASIS_ALL	anx_cc	Caudate	3	2.643	-	-
184	WP_GLP1_FROM_INTESTINE_AND_PANCREAS_AND_ROLE_IN_GLUCOSE_HOMEOSTASIS_ALL	anx_cc	Frontal Cortex BA9	4	2.468	-	-
185	WP_NAD_METABOLISM_SIRTUINS_AND_AGING_ALL	ptsd	Hippocampus	4	-2.324	-	-

**Table 9:** Differential Tests: Kruskal-Wallis and Pairwise Comparisons Note. MWU = Mann-Whitney U; RBC = rank-biserial correlation; ANX = anx_cc.

#	Gene Set	Comparison	Mean Z Diff	MWU p	FDR	Paired W p	RBC
1	WANG_ADIPOGENIC_GENES_REPRESSED_BY_SIRT1_ALL	PTSD vs ANX	-1.869	0.03538	0.03538	0.00769	4.14E-01
2	GOMF_NADPLUS_BINDING_ALL	PTSD vs ANX	-1.136	0.1131	0.1131	0.2633	0.322
3	HALLMARK_TNFA_SIGNALING_VIA_NFKB	PTSD vs ANX	-0.804	0.1167	0.1167	0.04421	2.92E-01
4	REACTOME_SIRT1_NEGATIVELY_REGULATES_RRNA_EXPRESSION_ALL	PTSD vs ANX	-1.210	0.3095	0.3095	0.5625	0.389
5	WP_GLP1_FROM_INTESTINE_AND_PANCREAS_AND_ROLE_IN_GLUCOSE_HOMEOSTASIS_ALL	PTSD vs ANX	-1.562	0.4857	0.4857	0.25	0.375

**Table 10:** Pairwise Disease Statistical Tests Note. d = Cohen's d; Perm = sign-flip permutation test; W = Wilcoxon signed-rank; ANX = anx_cc.

#	Gene Set	Comparison	n	Mean Diff	d (paired)	d (unpaired)	Significant Tests
1	WANG_ADIPOGENIC_GENES_REPRESSED_BY_SIRT1_ALL	PTSD vs ANX	18	-1.869	-0.74	-0.71	Welch p = .038; Paired t p = .006; Paired W p = .008; Perm p = .002
2	KEGG_NICOTINATE_AND_NICOTINAMIDE_METABOLISM_ALL	PTSD vs ANX	21	-1.121	-0.49	-0.73	Welch p = .022; Paired t p = .038; Perm p = .032
3	GOBP_CELLULAR_SENESCENCE_ALL	PTSD vs ANX	75	0.627	0.27	0.25	Paired t p = .024; Perm p = .026
4	HALLMARK_TNFA_SIGNALING_VIA_NFKB	PTSD vs ANX	20	-0.804	-0.51	-0.37	Paired t p = .034; Paired W p = .044; Perm p = .033
5	REACTOME_SIRT1_NEGATIVELY_REGULATES_RRNA_EXPRESSION_ALL	PTSD vs ANX	6	-1.210	-0.39	-0.53	|d (unpaired)| = 0.53
6	WP_GLP1_FROM_INTESTINE_AND_PANCREAS_AND_ROLE_IN_GLUCOSE_HOMEOSTASIS_ALL	PTSD vs ANX	4	-1.562	-1.32	-0.76	|d (paired)| = 1.32; |d (unpaired)| = 0.76
7	REACTOME_CYTOKINE_SIGNALING_IN_IMMUNE_SYSTEM	PTSD vs ANX	17	-0.748	-0.51	-0.52	|d (paired)| = 0.51; |d (unpaired)| = 0.52

**Table 11:** Concordance Metrics: Sign Rate, Lin's CCC, and Kendall τ Note. CCC = Lin's concordance correlation coefficient; τ = Kendall's tau; ANX = anx_cc.

#	Gene Set	Comparison	n	Sign Rate	Sign p	CCC	τ	τ p
1	MOOTHA_MITOCHONDRIA_ALL	PTSD vs ANX	353	0.646	0	0.503	2.81E-01	0
2	GOBP_INTRINSIC_APOPTOTIC_SIGNALING_PATHWAY_ALL	PTSD vs ANX	264	0.644	4.00E-06	0.465	0.311	0
3	HALLMARK_APOPTOSIS_ALL	PTSD vs ANX	131	0.677	8.10E-05	0.513	3.65E-01	0
4	GOBP_REGULATION_OF_SYNAPTIC_PLASTICITY_ALL	PTSD vs ANX	151	0.713	0	0.593	0.371	0
5	GOBP_CELLULAR_SENESCENCE_ALL	PTSD vs ANX	75	0.653	0.01058	0.57	0.387	1.00E-06
6	REACTOME_TELOMERE_ALL	PTSD vs ANX	54	0.641	0.05344	0.695	4.34E-01	4.00E-06
7	SAUL_SEN_MAYO_ALL	PTSD vs ANX	91	0.633	0.01487	0.475	0.312	1.20E-05
8	GOBP_REGULATION_OF_CELLULAR_SENESCENCE_ALL	PTSD vs ANX	35	0.743	0.005988	0.732	0.466	8.40E-05
9	HLA_COMPLEX_ALL	PTSD vs ANX	16	0.875	0.004181	0.869	6.67E-01	0.000135
10	REACTOME_REGULATION_OF_INSULIN_SECRETION_ALL	PTSD vs ANX	58	0.638	4.79E-02	0.479	0.302	0.000816
11	GOBP_INSULIN_SECRETION_INVOLVED_IN_CELLULAR_RESPONSE_TO_GLUCOSE_STIMULUS_ALL	PTSD vs ANX	53	0.627	9.19E-02	0.441	0.314	0.000895
12	KEGG_LTP_ALL	PTSD vs ANX	52	0.615	1.26E-01	0.472	3.11E-01	0.001149
13	GOCC_BCL_2_FAMILY_PROTEIN_COMPLEX_ALL	PTSD vs ANX	10	0.778	1.80E-01	0.762	6.89E-01	0.004687
14	ADULT_ASTRO_ALL	PTSD vs ANX	34	0.706	2.43E-02	0.446	0.312	0.009479
15	REACTOME_NEUROTRANSMITTER_RELEASE_CYCLE	PTSD vs ANX	11	0.727	2.27E-01	0.675	0.6	0.009946
16	ADULT_OLIGO_ALL	PTSD vs ANX	25	0.6	0.4244	0.722	0.353	0.01308
17	KEGG_DOPAMINERGIC_SYNAPSE	PTSD vs ANX	16	0.75	0.07681	0.601	0.433	0.01978
18	REACTOME_ACTIVATION_OF_INNATE_IMMUNE_RESPONSE	PTSD vs ANX	15	0.6	0.6072	0.672	0.391	0.04629
19	REACTOME_SYNTHESIS_SECRETION_AND_INACTIVATION_OF_GLP_1_ALL	PTSD vs ANX	12	0.667	0.3877	0.81	0.394	0.08632
20	REACTOME_MITOPHAGY	PTSD vs ANX	19	0.444	0.8145	0.62	0.275	0.1082
21	HALLMARK_INFLAMMATORY_RESPONSE	PTSD vs ANX	21	0.5	1	0.631	0.238	0.1403
22	GOBP_NAD_TRANSPORT_ALL	PTSD vs ANX	3	0.667	1	0.858	0.333	1
23	HALLMARK_TNFA_SIGNALING_VIA_NFKB	PTSD vs ANX	20	0.474	1	0.697	0.242	0.1458
24	REACTOME_CIRCADIAN_CLOCK	PTSD vs ANX	9	0.556	1	0.501	0.389	0.1802
25	WP_GLP1_FROM_INTESTINE_AND_PANCREAS_AND_ROLE_IN_GLUCOSE_HOMEOSTASIS_ALL	PTSD vs ANX	4	0.75	0.625	0.646	0.667	0.3333

**Table 12:** Gene-Level Leave-One-Out Influence: Flagged Genes Note. SF = sign-flip (✓ indicates removal of this gene flips the direction of the aggregate Stouffer Z); % = percentage influence on aggregate; Δ = change in Stouffer Z upon gene removal. Due to space constraints, gene sets with very large numbers of flagged genes (HALLMARK_OXIDATIVE_PHOSPHORYLATION ptsd, MONOAMINES ptsd, MOOTHA_MITOCHONDRIA ptsd, MOOTHA_PGC anx_cc/ptsd, REACTOME_CELLULAR_SENESCENCE/SENESCENCE ptsd) show the top influential genes with summary counts. Full gene-level data are available in the supplementary materials. Additional gene sets with fewer flagged genes (REACTOME_ACTIVATION_OF_INNATE_IMMUNE_RESPONSE, REACTOME_CIRCADIAN_CLOCK anx_cc, REACTOME_CYTOKINE_SIGNALING, REACTOME_ENERGY_DEPENDENT_REGULATION, REACTOME_GLP1, REACTOME_MITOPHAGY, REACTOME_NEUROTRANSMITTER_RELEASE_CYCLE, REACTOME_NICOTINATE_METABOLISM, REACTOME_SIRT1, REACTOME_SYNTHESIS_SECRETION_GLP1, SAUL_SEN_MAYO, WANG_ADIPOGENIC anx_cc, WP_GLP1, WP_NAD_BIOSYNTHETIC anx_cc, WP_NAD_METABOLISM, WP_NAD_METABOLISM_SIRTUINS, KEGG_MEDICUS_REFERENCE) are detailed in the supplementary files.

Gene-Set	Disease	Gene	Z	ΔStouffer	%	SF
ADULT_ASTRO_ALL	anx_cc	GRM3	7.36	+1.30	91.3	-
		ITGA7	-5.42	-0.92	64.8	-
		SDC4	-4.81	-0.82	57.2	-
		SLC39A12	3.64	+0.66	46.0	-
		CLTCL1	-3.80	-0.64	44.9	-
		PRODH	-3.38	-0.57	39.7	-
		ACSBG1	3.05	+0.55	38.8	-
		AGT	-3.13	-0.52	36.7	-
		ITGB4	2.69	+0.49	34.3	-
		GFAP	-2.79	-0.46	32.5	-
		SLC25A18	2.21	+0.41	28.5	-
		SPARCL1	-2.24	-0.37	25.9	-
		SLC14A1	1.95	+0.36	25.4	-
ADULT_ASTRO_ALL	ptsd	RNF43	-5.03	-0.83	24.7	-
ADULT_MICRO_ALL	anx_cc	TREM1	5.54	+1.21	122.2	✓
		CD83	-3.02	-0.70	70.4	-
		RGS10	3.00	+0.65	65.0	-
		CD74	-2.75	-0.64	64.2	-
		A2M	1.94	+0.41	41.1	-
		CD14	-1.67	-0.40	40.0	-
		ITGAX	-1.40	-0.34	34.0	-
		GALR1	1.19	+0.24	24.3	-
		LAPTM5	1.17	+0.24	23.9	-
ADULT_MICRO_ALL	ptsd	OLR1	-6.24	-1.43	97.0	-
		RGS10	-3.60	-0.81	54.9	-
		CD83	2.92	+0.73	49.5	-
		HLA-DRA	2.74	+0.69	46.6	-
		ST8SIA4	2.04	+0.52	35.4	-
		CD14	-2.31	-0.51	34.3	-
		C3	1.19	+0.32	21.8	-
ADULT_OLIGO_ALL	anx_cc	GLYCTK	11.94	+2.35	57.2	-
		PLEKHB1	7.99	+1.55	37.6	-
ADULT_OLIGO_ALL	ptsd	GLYCTK	8.73	+1.67	31.6	-
		PLEKHB1	7.77	+1.48	27.9	-
GOBP_CAMKK_AMPK_SIGNALING_CASCADE_ALL	anx_cc	LRRK2	-2.70	-0.74	25.9	-
		ZMPSTE24	1.68	+0.71	24.9	-
GOBP_CAMKK_AMPK_SIGNALING_CASCADE_ALL	ptsd	LRRK2	-4.60	-1.54	104.1	✓
		TREM2	3.72	+1.40	95.1	-
		KCNJ11	-3.61	-1.19	80.5	-
		ZMPSTE24	1.98	+0.79	53.6	-
		MYH7B	1.95	+0.78	52.9	-
		PCP4	-1.96	-0.60	40.9	-
GOBP_CELLULAR_SENESCENCE_ALL	anx_cc	NEK4	12.93	+1.48	100.5	-
		FBXO4	-6.84	-0.77	52.2	-
		YPEL3	-5.95	-0.67	45.3	-
		PNPT1	-5.21	-0.58	39.6	-
		OPA1	-4.78	-0.54	36.3	-
		ZMIZ1	-4.59	-0.51	34.8	-
		NPM1	4.39	+0.51	34.5	-
		HLA-G	4.26	+0.50	33.6	-
		MAPK14	-4.15	-0.46	31.4	-
		SMC6	-4.13	-0.46	31.2	-
		SPI1	3.77	+0.44	29.7	-
		HRAS	3.44	+0.40	27.2	-
		TBX2	3.37	+0.39	26.7	-
		MAPK10	-3.47	-0.39	26.1	-
		MAPK9	-3.33	-0.37	25.1	-
		NEK6	3.13	+0.37	24.8	-
		ING2	-3.18	-0.35	23.9	-
		PRELP	-3.13	-0.35	23.5	-
		PML	2.52	+0.30	20.1	-
		BMPR1A	2.51	+0.30	20.1	-
GOBP_CELLULAR_SENESCENCE_ALL	ptsd	NEK4	7.80	+0.87	21.6	-
GOBP_GLUTAMATERGIC_ALL	anx_cc	GRM3	7.36	+0.82	87.6	-
		GRIK2	-5.67	-0.64	68.6	-
		GRIK5	-4.01	-0.45	48.7	-
		GRIA2	3.89	+0.43	46.0	-
		CLCN3	3.66	+0.40	43.3	-
		SLC1A4	-3.39	-0.39	41.3	-
		SERPINE2	3.49	+0.38	41.2	-
		NTRK1	3.32	+0.37	39.2	-
		VPS54	-3.08	-0.35	37.5	-
		P2RX1	-2.98	-0.34	36.4	-
		TNF	3.00	+0.33	35.3	-
		ATP1A2	-2.79	-0.32	34.1	-
		LRRK2	-2.70	-0.31	32.9	-
		HOMER1	-2.59	-0.30	31.7	-
		HCN1	2.38	+0.26	27.9	-
		ADORA1	2.32	+0.25	27.2	-
		GRIN1	2.31	+0.25	27.1	-
		GRID1	2.27	+0.25	26.6	-
		DRD2	-2.16	-0.25	26.6	-
		CDH8	-2.15	-0.25	26.4	-
		CNIH2	-2.14	-0.25	26.3	-
		ADORA2A	2.22	+0.24	26.0	-
		ATAD1	-2.04	-0.23	25.1	-
		DTNBP1	-2.04	-0.23	25.0	-
		GRIA4	-1.96	-0.23	24.1	-
		UNC13C	1.99	+0.22	23.2	-
		NAPA	1.91	+0.21	22.3	-
		PSEN1	1.86	+0.20	21.7	-
		GRM2	1.85	+0.20	21.6	-
		SLC17A6	-1.72	-0.20	21.3	-
GOBP_GLUTAMATERGIC_ALL	ptsd	ABCC8	6.47	+0.74	54.1	-
		GRIA1	5.35	+0.61	44.6	-
		RAB3GAP1	-4.89	-0.57	42.1	-
		LRRK2	-4.60	-0.54	39.6	-
		SLC17A7	4.35	+0.49	36.1	-
		CACNB4	-4.12	-0.48	35.5	-
		GRIK2	-3.88	-0.46	33.5	-
		FRRS1L	-3.44	-0.41	29.8	-
		CNIH2	3.55	+0.40	29.4	-
		HOMER3	-3.22	-0.38	27.9	-
		DISC1	-3.19	-0.38	27.6	-
		DRD2	-3.08	-0.36	26.7	-
		CACNG5	2.87	+0.32	23.6	-
		SYT1	2.66	+0.30	21.8	-
		HCN1	2.57	+0.29	21.1	-
		RELN	2.57	+0.29	21.1	-
		GRIA2	2.56	+0.29	21.0	-
		CLCN3	2.53	+0.28	20.8	-
GOBP_INSULIN_SECRETION…GLUCOSE_ALL	anx_cc	GPR27	9.92	+1.31	25.0	-
GOBP_INSULIN_SECRETION…GLUCOSE_ALL	ptsd	EFNA5	6.90	+0.91	20.0	-
GOBP_INTRINSIC_APOPTOTIC_SIGNALING_PATHWAY_ALL	ptsd	GPX1	10.78	+0.66	32.9	-
		NBN	7.43	+0.45	22.6	-
GOBP_POSITIVE_REGULATION_OF_MICROGLIAL_ACTIVATION	anx_cc	SPP1	-5.94	-1.52	343.0	✓
		AIF1	5.32	+1.39	313.2	-
		TNF	3.00	+0.79	177.8	-
		APOE	2.78	+0.73	165.2	-
		TLR4	2.63	+0.69	156.4	-
		P2RY12	-2.45	-0.62	139.2	✓
		TREM2	-1.85	-0.46	104.4	✓
		TLR2	-1.58	-0.39	88.8	-
		CD68	-1.52	-0.38	85.3	-
		IL6	1.36	+0.37	82.6	-
		ITGAX	-1.40	-0.35	78.4	-
		CX3CR1	-1.18	-0.29	65.4	-
		CX3CL1	-0.55	-0.13	28.6	-
		IL1B	0.33	+0.10	22.3	-
		ITGAM	-0.41	-0.09	20.4	-
GOBP_POSITIVE_REGULATION_OF_MICROGLIAL_ACTIVATION	ptsd	AIF1	4.94	+1.19	44.8	-
		TREM2	3.72	+0.87	32.9	-
GOBP_REGULATION_OF_CELLULAR_SENESCENCE_ALL	anx_cc	NEK4	12.93	+2.15	97.8	-
		YPEL3	-5.95	-1.04	47.1	-
		PNPT1	-5.21	-0.91	41.4	-
		HLA-G	4.26	+0.69	31.3	-
		ING2	-3.18	-0.57	25.8	-
		TBX2	3.37	+0.54	24.5	-
		NEK6	3.13	+0.50	22.6	-
		EEF1E1	-2.59	-0.47	21.3	-
GOBP_REGULATION_OF_CELLULAR_SENESCENCE_ALL	ptsd	NEK4	7.80	+1.30	56.0	-
		TERF2	-3.61	-0.65	28.0	-
		EEF1E1	-3.45	-0.63	26.9	-
		ZKSCAN3	3.81	+0.62	26.6	-
		YPEL3	-3.32	-0.60	25.9	-
		MORC3	3.52	+0.57	24.4	-
		HLA-G	3.46	+0.56	24.0	-
		NEK6	3.27	+0.53	22.6	-
		MIF	-2.75	-0.51	21.7	-
		PLK2	-2.70	-0.50	21.4	-
GOBP_REGULATION_OF_SYNAPTIC_PLASTICITY_ALL	anx_cc	MAP1A	-7.33	-0.59	36.1	-
		STAU1	-7.09	-0.57	35.0	-
		VAMP2	6.98	+0.55	33.8	-
		PICK1	6.09	+0.48	29.4	-
		STX4	5.83	+0.46	28.2	-
		GRIK2	-5.67	-0.46	28.0	-
		PRNP	-5.55	-0.45	27.4	-
		ARC	-4.64	-0.38	23.0	-
		EIF2AK4	-4.47	-0.36	22.2	-
		SORCS3	-4.43	-0.36	21.9	-
		GSK3B	-4.40	-0.36	21.8	-
GOBP_REGULATION_OF_SYNAPTIC_PLASTICITY_ALL	ptsd	MAP1A	-10.51	-0.84	60.3	-
		STAU1	-5.91	-0.47	33.8	-
		GRIA1	5.35	+0.44	31.2	-
		EIF2AK4	-5.00	-0.40	28.5	-
		AGER	4.84	+0.40	28.2	-
		RAB3GAP1	-4.89	-0.39	27.9	-
		STX4	4.71	+0.39	27.5	-
		PRNP	-4.77	-0.38	27.2	-
		SORCS2	-4.31	-0.34	24.5	-
		SLC8A2	3.93	+0.32	23.0	-
		DLG4	-3.96	-0.32	22.5	-
		GRIK2	-3.88	-0.31	22.1	-
		ACE	3.74	+0.31	21.9	-
		VAMP2	3.57	+0.29	20.9	-
		S100B	-3.67	-0.29	20.9	-
		HRAS	3.51	+0.29	20.6	-
		ITPKA	-3.62	-0.29	20.6	-
		C22ORF39	3.42	+0.28	20.0	-
GOBP_SYNAPSE_PRUNING_ALL	anx_cc	EPHA4	-3.26	-0.84	98.9	-
		SARM1	3.10	+0.75	88.0	-
		PLXNC1	2.73	+0.66	77.1	-
		C1QC	2.47	+0.59	69.4	-
		TREM2	-1.85	-0.49	57.4	-
		VANGL2	1.64	+0.38	45.1	-
		C1QA	1.53	+0.36	41.8	-
		KCNK13	-1.32	-0.36	41.8	-
		NGEF	-1.25	-0.34	39.9	-
		CX3CR1	-1.18	-0.32	37.7	-
GOBP_SYNAPSE_PRUNING_ALL	ptsd	TREM2	3.72	+0.86	36.2	-
		C1QA	-2.84	-0.78	33.1	-
		VANGL2	-2.60	-0.72	30.6	-
		SARM1	3.15	+0.71	30.2	-
		PLXNC1	3.10	+0.70	29.7	-
GOCC_BCL_2_FAMILY_PROTEIN_COMPLEX_ALL	anx_cc	BAK1	-3.92	-1.38	98.9	-
		BIK	2.97	+0.91	65.4	-
		BCL2L1	2.82	+0.86	61.9	-
		BAD	2.67	+0.81	58.2	-
		BCL2	-1.18	-0.47	33.5	-
		BCL2L11	-0.79	-0.34	24.2	-
		MCL1	1.21	+0.33	23.4	-
GOCC_BCL_2_FAMILY_PROTEIN_COMPLEX_ALL	ptsd	BIK	5.15	+1.54	47.8	-
		BAK1	-1.94	-0.82	25.4	-
		BCL2L2	2.87	+0.78	24.3	-
GOMF_NADPLUS_BINDING_ALL	anx_cc	HADH	-5.76	-1.51	68.8	-
		SIRT3	3.20	+0.73	33.4	-
		ZC3HAV1	3.15	+0.72	32.9	-
		SIRT5	2.84	+0.64	29.3	-
		PARP15	2.55	+0.57	26.0	-
GOMF_NADPLUS_BINDING_ALL	ptsd	SIRT3	-7.58	-1.82	72.9	-
		GLUD1	-3.72	-0.85	34.2	-
		SIRT2	3.10	+0.85	34.1	-
		PARP9	2.04	+0.59	23.5	-
HALLMARK_APOPTOSIS_ALL	anx_cc	MGMT	-10.19	-0.89	23.0	-
HALLMARK_APOPTOSIS_ALL	ptsd	GPX1	10.78	+0.93	31.8	-
HALLMARK_INFLAMMATORY_RESPONSE	anx_cc	NOS2	8.24	+1.73	36.8	-
		STAT1	-3.73	-0.95	20.2	-
HALLMARK_INFLAMMATORY_RESPONSE	ptsd	NOS2	7.71	+1.70	183.9	✓
		IL1A	-4.11	-0.94	101.8	-
		CCL5	3.09	+0.67	72.3	-
		VCAM1	-2.30	-0.54	58.1	-
		STAT1	-1.66	-0.39	42.5	-
		CCL2	-1.46	-0.35	37.8	-
		TNF	1.56	+0.33	35.3	-
		FOS	-1.28	-0.31	33.5	-
		STAT3	1.47	+0.31	33.1	-
		IL1B	-1.03	-0.25	27.5	-
		PTGS2	-0.82	-0.21	22.4	-
HALLMARK_OXIDATIVE_PHOSPHORYLATION_ALL	anx_cc	NDUFC2	7.94	+0.64	22.9	-
		SLC25A12	7.03	+0.56	20.3	-
HALLMARK_OXIDATIVE_PHOSPHORYLATION_ALL	ptsd	54 genes flagged (top: NDUFA2, Z = 9.80, Δ = +0.79, 95.0%; RHOT1, Z = 9.12, Δ = +0.74, 88.4%; see full listing)				
HALLMARK_PI3K_AKT_MTOR_SIGNALING_ALL	anx_cc	UBE2D3	8.21	+0.89	51.5	-
		YWHAB	7.83	+0.84	49.1	-
		DDIT3	4.61	+0.50	29.2	-
		NFKBIB	4.30	+0.47	27.2	-
		GSK3B	-4.40	-0.46	26.7	-
		PLA2G12A	4.05	+0.44	25.7	-
		RAC1	3.61	+0.40	23.0	-
		RAF1	-3.71	-0.39	22.5	-
		NCK1	-3.67	-0.38	22.2	-
		HRAS	3.44	+0.38	21.9	-
		CDK4	-3.54	-0.37	21.4	-
		PAK4	-3.54	-0.37	21.4	-
		MAPK10	-3.47	-0.36	20.9	-
		MAPK9	-3.33	-0.35	20.1	-
HALLMARK_PI3K_AKT_MTOR_SIGNALING_ALL	ptsd	UBE2D3	8.52	+0.91	69.5	-
		ECSIT	4.72	+0.50	38.2	-
		MAPKAP1	4.38	+0.46	35.5	-
		GRB2	4.19	+0.44	33.9	-
		RIPK1	-3.95	-0.43	33.1	-
		CLTC	-3.94	-0.43	33.0	-
		IRAK4	4.01	+0.42	32.4	-
		PDK1	3.83	+0.41	30.9	-
		ADCY2	3.64	+0.38	29.4	-
		NCK1	3.60	+0.38	29.0	-
		HRAS	3.51	+0.37	28.3	-
		E2F1	3.30	+0.35	26.6	-
		PPP2R1B	-2.97	-0.33	25.0	-
		MKNK1	2.81	+0.30	22.6	-
		TRIB3	-2.67	-0.30	22.5	-
		CDK4	-2.62	-0.29	22.1	-
		ITPR2	2.67	+0.28	21.4	-
		DDIT3	2.60	+0.27	20.8	-
HALLMARK_TNFA_SIGNALING_VIA_NFKB	anx_cc	NOS2	8.24	+1.77	38.3	-
HALLMARK_TNFA_SIGNALING_VIA_NFKB	ptsd	NOS2	7.71	+1.74	170.1	✓
		CCL5	3.09	+0.68	66.7	-
		VCAM1	-2.30	-0.55	54.2	-
		CHUK	-1.93	-0.47	45.8	-
		CCL2	-1.46	-0.36	35.4	-
		TNF	1.56	+0.33	32.4	-
		FOS	-1.28	-0.32	31.4	-
		IL1B	-1.03	-0.26	25.8	-
		EGR1	1.21	+0.25	24.4	-
		CXCL8	-0.97	-0.25	24.4	-
		ATF3	-0.93	-0.24	23.4	-
		PTGS2	-0.82	-0.22	21.0	-
HLA_COMPLEX_ALL	anx_cc	HLA-DMA	-7.66	-1.84	45.3	-
		HLA-DMB	-7.11	-1.70	41.8	-
		HLA-DOB	-5.94	-1.40	34.4	-
		HLA-G	4.26	+1.23	30.3	-
		HLA-DPA1	3.00	+0.91	22.3	-
HLA_COMPLEX_ALL	ptsd	HLA-DMA	-9.18	-2.18	58.6	-
		HLA-C	-8.04	-1.89	51.0	-
		HLA-DOB	-6.00	-1.38	37.2	-
		HLA-DMB	-5.77	-1.33	35.8	-
		HLA-B	3.73	+1.05	28.2	-
		HLA-DQB2	3.69	+1.04	27.9	-
		HLA-G	3.46	+0.98	26.3	-
		HLA-DRA	2.74	+0.80	21.5	-
KEGG_APOPTOSIS_ALL	anx_cc	CASP10	6.40	+0.75	20.6	-
KEGG_APOPTOSIS_ALL	ptsd	ATM	6.30	+0.76	48.8	-
		PPP3CA	-4.84	-0.60	38.9	-
		CASP7	4.84	+0.58	37.3	-
		DFFB	-4.22	-0.53	33.9	-
		IL1A	-4.11	-0.51	33.1	-
		RIPK1	-3.95	-0.49	31.9	-
		CAPN1	4.04	+0.48	31.0	-
		IRAK4	4.01	+0.48	30.8	-
		CASP9	-3.74	-0.47	30.1	-
		CASP6	3.23	+0.38	24.6	-
		IRAK2	3.01	+0.36	22.9	-
		PPP3R1	2.94	+0.35	22.4	-
		PIK3CB	2.68	+0.32	20.4	-
KEGG_DOPAMINERGIC_SYNAPSE	anx_cc	VAMP2	6.98	+1.74	95.3	-
		PPP3CA	-4.65	-1.26	69.0	-
		PPP2CA	3.96	+0.96	52.6	-
		STX1A	-3.34	-0.92	50.4	-
		PPP2CB	2.67	+0.63	34.4	-
		DRD2	-2.16	-0.62	33.8	-
		DRD4	2.17	+0.50	27.4	-
		DRD5	-1.65	-0.49	26.6	-
KEGG_DOPAMINERGIC_SYNAPSE	ptsd	STX1A	-7.01	-1.77	145.8	✓
		TH	6.18	+1.64	134.8	-
		PPP3CA	-4.84	-1.21	99.8	-
		VAMP2	3.57	+0.96	79.2	-
		PPP1R1B	3.26	+0.88	72.5	-
		DRD2	-3.08	-0.76	62.2	-
		PPP2CB	-2.86	-0.70	57.6	-
		SYN2	2.24	+0.62	50.9	-
		DRD4	1.90	+0.53	43.6	-
		DRD3	-1.76	-0.41	34.2	-
		DRD5	-1.59	-0.37	30.6	-
		DDC	-1.58	-0.37	30.4	-
		PPP2CA	1.20	+0.35	28.9	-
KEGG_LTP_ALL	anx_cc	EP300	7.55	+1.00	41.8	-
		PPP1CB	6.28	+0.83	34.6	-
		PPP1CC	-5.30	-0.74	30.8	-
		MAPK3	5.37	+0.70	29.4	-
		PPP3CA	-4.65	-0.65	27.2	-
		PLCB3	-4.14	-0.58	24.3	-
		RAF1	-3.71	-0.52	21.9	-
		GRIA2	3.89	+0.50	21.1	-
KEGG_LTP_ALL	ptsd	EP300	5.76	+0.78	34.2	-
		GRIA1	5.35	+0.73	31.7	-
		PPP3CA	-4.84	-0.70	30.6	-
		HRAS	3.51	+0.47	20.5	-
		GNAQ	-3.19	-0.47	20.5	-
KEGG_NICOTINATE_AND_NICOTINAMIDE_METABOLISM_ALL	anx_cc	NT5C3A	4.41	+0.86	20.6	-
KEGG_NICOTINATE_AND_NICOTINAMIDE_METABOLISM_ALL	ptsd	PNP	-4.62	-0.99	58.8	-
		NUDT12	2.82	+0.67	39.9	-
		NMNAT2	2.62	+0.63	37.1	-
		QPRT	-2.63	-0.55	32.4	-
		NT5C2	-2.36	-0.49	28.8	-
		NT5E	-2.26	-0.46	27.4	-
		NT5C	-1.95	-0.39	23.3	-
		NT5C3A	1.35	+0.34	20.4	-
MONOAMINES_ALL	anx_cc	VAMP2	6.98	+0.82	26.4	-
MONOAMINES_ALL	ptsd	44 genes flagged (top: GNB2, Z = -10.69, Δ = -1.29, 250.8%; STX1A, Z = -7.01, Δ = -0.85, 164.7%; see full listing)				
MOOTHA_MITOCHONDRIA_ALL	anx_cc	CD40	-13.52	-0.72	24.4	-
MOOTHA_MITOCHONDRIA_ALL	ptsd	21 genes flagged (top: CD40, Z = -14.72, Δ = -0.78, 55.1%; NDUFA2, Z = 9.80, Δ = +0.52, 36.5%)				
MOOTHA_PGC_ALL	anx_cc	99-genes-flagged-(top:-S100A1,-Z-=-8.95,-Δ-=-+0.49,-81.5%;-MTCH2,-Z-=--8.63,-Δ-=--0.47,-78.3%)				
MOOTHA_PGC_ALL	ptsd	82 genes flagged (top: LSM8, Z = 8.65, Δ = +0.48, 73.8%; PSMB7, Z = -7.44, Δ = -0.41, 63.1%)				
REACTOME_CELLULAR_SENESCENCE_ALL-/-SENESCENCE_ALL	ptsd	50 genes flagged (top: NBN, Z = 7.43, Δ = +0.74, 115.1% SF; MOV10, Z = -7.15, Δ = -0.72, 111.7%)				
REACTOME_CIRCADIAN_CLOCK	ptsd	CLOCK	7.09	+2.33	78.5	-
		NPAS2	-3.12	-1.28	43.4	-
		CRY2	-2.60	-1.10	37.1	-
		ARNTL	3.06	+0.90	30.5	-
		NR1D1	2.82	+0.82	27.6	-
REACTOME_COMPLEMENT_ALL	anx_cc	C4A	11.01	+1.72	58.2	-
		CD55	-3.55	-0.61	20.4	-
REACTOME_COMPLEMENT_ALL	ptsd	C4A	12.33	+2.08	79.1	-
		CD46	8.85	+1.48	56.3	-
		CD81	4.35	+0.71	27.0	-
		FCN3	-3.07	-0.57	21.6	-
		C5	-3.04	-0.56	21.4	-
		C1QA	-2.84	-0.53	20.0	-
REACTOME_REGULATION_OF_INSULIN_SECRETION_ALL	anx_cc	CACNA2D2	-11.05	-1.40	44.0	-
		VAMP2	6.98	+0.93	29.2	-
REACTOME_REGULATION_OF_INSULIN_SECRETION_ALL	ptsd	CACNA2D2	-12.79	-1.64	26.5	-
		GNB2	-10.69	-1.36	22.0	-
REACTOME_TELOMERE_ALL	anx_cc	PPP6C	9.25	+1.23	41.5	-
		RPA3	-5.66	-0.80	26.9	-
		RUVBL1	-5.59	-0.79	26.6	-
		RFC2	-4.91	-0.70	23.4	-
		RUVBL2	4.62	+0.60	20.2	-
REACTOME_TELOMERE_ALL	ptsd	PPP6C	11.46	+1.56	90.4	-
		POT1	7.00	+0.94	54.9	-
		RPA3	-6.28	-0.88	51.0	-
		RFC2	-5.45	-0.77	44.4	-
		TERF1	4.72	+0.63	36.8	-
		POLA2	-4.10	-0.58	33.6	-
		NOP10	4.18	+0.56	32.4	-
		TEN1	-3.62	-0.51	29.8	-
		DSCC1	-3.62	-0.51	29.8	-
		TERF2	-3.61	-0.51	29.7	-
		PCNA	-3.29	-0.47	27.2	-
		WRN	3.50	+0.47	27.0	-
		RTEL1	3.44	+0.46	26.5	-
		PIF1	3.20	+0.42	24.6	-
		POLR2J	-2.77	-0.40	23.0	-
		RUVBL2	2.69	+0.35	20.5	-
WANG_ADIPOGENIC_GENES_REPRESSED_BY_SIRT1_ALL	ptsd	HIGD1A	-7.59	-1.70	34.0	-
		OLR1	-6.24	-1.37	27.4	-
WP_NAD_BIOSYNTHETIC_PATHWAYS_ALL	ptsd	TNKS	-10.13	-2.23	37.9	-
		SIRT3	-7.58	-1.62	27.6	-
